# The affinity and selectivity of α‐adrenoceptor antagonists, antidepressants, and antipsychotics for the human α1A, α1B, and α1D‐adrenoceptors

**DOI:** 10.1002/prp2.602

**Published:** 2020-07-01

**Authors:** Richard G. W. Proudman, Andre S. Pupo, Jillian G. Baker

**Affiliations:** ^1^ Cell Signalling Research Group Division of Physiology, Pharmacology and Neuroscience School of Life Sciences C Floor Medical School Queen’s Medical Centre University of Nottingham Nottingham UK; ^2^ Department of Pharmacology Institute of Biosciences São Paulo State University Botucatu‐São Paulo Brazil

**Keywords:** affinity, antidepressant, antipsychotic, benign prostatic hypertrophy, hypertension, α antagonist

## Abstract

α1‐adrenoceptor antagonists are widely used for hypertension (eg, doxazosin) and benign prostatic hypertrophy (BPH, eg, tamsulosin). Some antidepressants and antipsychotics have been reported to have α1 affinity. This study examined 101 clinical drugs and laboratory compounds to build a comprehensive understanding of α1‐adrenoceptor subtype affinity and selectivity. [3H]prazosin whole‐cell binding was conducted in CHO cells stably expressing either the full‐length human α1A, α1B, or α1D‐adrenoceptor. As expected, doxazosin was a high‐affinity nonselective α1‐antagonist although other compounds (eg, cyclazosin, 3‐MPPI, and ARC239) had higher affinities. Several highly α1A‐selective antagonists were confirmed (SNAP5089 had over 1700‐fold α1A selectivity). Despite all compounds demonstrating α1 affinity, only BMY7378 had α1D selectivity and no α1B‐selective compounds were identified. Phenoxybenzamine (used in pheochromocytoma) and dibenamine had two‐component‐binding inhibition curves at all three receptors. Incubation with sodium thiosulfate abolished the high‐affinity component suggesting this part is receptor mediated. Drugs used for hypertension and BPH had very similar α1A/α1B/α1D‐adrenoceptor pharmacological profiles. Selective serotonin reuptake inhibitors (antidepressants) had poor α1‐adrenoceptor affinity. Several tricyclic antidepressants (eg, amitriptyline) and antipsychotics (eg, chlorpromazine and risperidone) had high α1‐adrenoceptor affinities, similar to, or higher than, α blockers prescribed for hypertension and BPH, whereas others had poor α1 affinity (eg, protriptyline, sulpiride, amisulpiride, and olanzapine). The addition of α blockers for the management of hypertension or BPH in people already taking tricyclic antidepressants and certain antipsychotics may not be beneficial. Awareness of the α‐blocking potential of different antipsychotics may affect the choice of drug for those with delirium where additional hypotension (eg, in sepsis) may be detrimental.

**Abbreviations**
BPH: benign prostatic hypertrophy
CHO: Chinese hamster ovary
SSRI: selective serotonin reuptake inhibitor
TCA: tricyclic antidepressant

## 1 INTRODUCTION

The α1‐adrenoceptors are expressed in a wide range of tissues including blood vessels, kidney, spleen, liver, brain, and lower urinary tract.^1^, ^2^, ^3^ There are three subtypes: α1A, α1B, and α1D‐adrenoceptors.^1^, ^2^, ^3^, ^4^ All are present in blood vessels, and whilst α1A and α1D and are both important in smooth muscle contraction (and control of blood pressure), the role of the α1B‐adrenoceptors is less certain.^2^, ^3^, ^5^, ^6^

α‐adrenoceptor antagonists (α blockers) were first used to reduce systemic blood pressure with dibenamine, phentolamine, and phenoxybenzamine used in the diagnosis and management of pheochromocytoma, an adrenal catecholamine‐secreting tumor.^7^, ^8^ While phenoxybenzamine is still important for pheochromocytoma, longer acting, nonselective α1‐antagonists were developed (doxazosin, terazosin, indoramin, and prazosin) and remain important in the management of resistant hypertension.

α blockers are also used in benign prostatic hypertrophy (BPH) where α1A blockade induces prostate and lower urinary tract smooth muscle relaxation, improving urinary flow.^9^ Phenoxybenzamine was the first α blocker to be used in BPH^10^ although its α2 effects limited its use.^11^ The nonselective α1‐antagonists doxazosin, terazosin, indoramin, and prazosin were used effectively for BPH, but caused hypotension, particularly postural hypotension, and required dose titration to manage this problematic side effect.^9^, ^12^ Selective α1A‐antagonists were developed, hoping to minimize hypotension by reducing α1B‐antagonsim.^11^, ^13^ Tamsulosin, alfuzosin, and silodosin were developed as prostate‐specific (α1A selective) drugs and are used without dose titration.^9^ Despite reports of “better tolerability,”^11^, ^14^ alfuzosin is reported to be a nonselective α1‐antagonist and tamsulosin to have equal α1A‐ and α1D‐adrenoceptor affinity,^15^, ^16^ suggesting they may be pharmacologically indistinguishable from drugs used for hypertension. Indeed, tamsulosin (the most commonly prescribed α blocker for BPH) is associated with increased hypotension, falls, and fractures.^12^, ^13^, ^17^ Although effective for BPH, silodosin appears to have more sexual side effects, whereas its cardiovascular effects remain uncertain.^18^

α1‐adrenoceptors are the most abundant adrenoceptors in the brain and modulate neurotransmitter release.^3^ Many antidepressants prevent the reuptake of neurotransmitters (serotonin and noradrenaline), and therefore increase synaptic neurotransmitter concentration. However, several antidepressants have significant α1‐adrenoceptor affinity.^19^, ^20^, ^21^ This high affinity is seen in brain homogenates.^22^ In theory these two effects (increased neurotransmitter presence, but receptor blockade) could cancel each other out.^20^ However, antidepressants cause hypotension, particularly postural hypotension (up to 58% users^23^, ^24^). Not surprisingly therefore, antidepressant use is associated with twice the risk of falls.^25^

Several antipsychotics (neuroleptics) bind to α1‐adrenoceptors in blood vessels and brain homogenates.^6^, ^26^, ^27^ Many antipsychotics cause postural hypotension,^28^, ^29^ and again, rates are high (eg, 48% taking risperidone^24^) including postural hypotension in those taking long‐term antipsychotics (77%^30^). Interestingly, the degree of postural hypotension seen with several antipsychotics correlates well with the α1A‐adrenoceptor affinity.^29^ Antipsychotic drug use is also associated with falls and hip fractures and regular use is associated with twice the risk of falls (even after controlling for other risks^31^, ^32^).

There are many studies examining the affinity of α1‐adrenoceptor ligands. Many are older studies before the identification of the three subtypes and many are in whole tissue where multiple subtypes will be present. Most studies only report the two or three ligands under investigation. Here we aimed to investigate the subtype selectivity of a wide range of α‐antagonists including those used in hypertension, BPH, antidepressants, antipsychotics as well as laboratory compounds. Human α1A, α1B, and α1D‐adrenoceptors were expressed in intact mammalian cells, in order to build a comprehensive and directly comparable picture of α1‐subtype selectivity in living cells.

## 2 METHODS

### 2.1 Materials

A list of all of the compounds studied, together with the source and supplier code from which it was purchased, is given in Table S1. White‐sided view plates were from Greiner Bio‐one, Kremsmunster, Austria; and [3H]prazosin, Microscint 20, and scintillation fluid from PerkinElmer (Buckinghamshire, UK). Fetal calf serum was from Gibco (Thermo‐Fisher), Lipofectamine, and OPTIMEM were from Life Technologies, Thermo‐Fisher, Massachusetts USA. All other cell culture reagents were from Sigma Chemicals (Poole, Dorset, UK).

### 2.2 Cell lines

CHO‐K1 (RIDD: CVCL_0214) were stably transfected with the DNA of the human α1A‐adrenoceptor, human α1B‐adrenoceptor (DNAs from Guthrie DNA Resource Centre), or human α1D‐adrenoceptor (full‐length DNA from Andre Pupo^33^; using Lipofectaime and Optimem according to the manufacturers’ instructions. Transfected cells were selected for 3 weeks using resistance to neomycin (at 1mg/ml). Single clones from each transfection were then isolated by dilution cloning giving rise to the stable cell lines CHO‐α1A, CHO‐α1B, and CHO‐α1D.

### 2.3 Cell culture

CHO cells were grown in Dulbecco's modified Eagle's medium nutrient mix F12 (DMEM/F12) containing 10% fetal calf serum and 2 mmol/L L‐glutamine in a 37°C humidified 5% CO_2_: 95% air atmosphere. Cells were seeded into white‐sided, clear bottomed 96‐well view plates and grown to confluence.

### 2.4 [3H]prazosin binding—saturation binding

The K_D_ value for [3H]prazosin was determined in each cell line by saturation binding. [3H]prazosin was diluted in serum‐free media. Media was removed from each well and replaced with either 100 µL serum‐free media (total binding) or 100 µL 20 µmol/L tamsulosin (α1A and α1B) or 200 µmol/L tamsulosin (α1D) to determine nonspecific binding. [3H]prazosin was then added to the wells (quadruplicates per condition, 1 in 2 dilution in well), and the plates incubated for 2 hour at 37°C in a humidified 5% CO_2_: 95% air atmosphere. After 2 hours, the cells were washed twice by the addition and removal of 2 × 200 µL cold (4°C) phosphate‐buffered saline. 100 µL Microscint 20 was added to each well and a white base applied to the plate to convert the wells into white‐sided/white‐bottomed wells. Plates were left at room temperature for at least 6 hours before being counted on a Topcount (PerkinElmer), with a counting time of 2 minutes per well.

### 2.5 [3H]prazosin whole‐cell binding—competition binding

Ligands were serially diluted in serum‐free media (DMEM/F12 containing 2 mmol/L L‐glutamine only) to twice their final required concentration. Media were removed from each well of the 96‐well view plate and 100 µL ligand added to triplicate wells. This was immediately followed by the addition of 100 µL [3H]prazosin (diluted in serum‐free media) and the cells incubated for 2 hours at 37°C (5% CO_2_, humidified atmosphere). After 2 hours the plates were washed as above. Cells were inspected under a light microscope to ensure cells were still present after the wash and before the addition of Microscint 20. In a few cases, high concentrations of competing ligand caused the cells to round up and be washed off the plates. These concentrations were excluded from the analysis. Total binding (6 wells/plate) and nonspecific binding (6 wells/plate) determined by the presence of 10 µmol/L tamsulosin (α1A and α1B) or 100 µmol/L tamsulosin (α1D) was defined in every plate.

Sodium thiosulfate reacts with 2‐chloroethylamines in a 1:1 stoichiometry to inactivate the ethyleniminium ions generated in solution (see Discussion). Sodium thiosulfate had no effect on [3H]prazosin binding up to concentrations of 10 mmol/L. Therefore, to ensure that all ethyleniminium ions were inactivated, sodium thiosulfate was used in excess, with a final well concentration of 1 mmol/L. When used, competing ligands were serially diluted in serum‐free media (just as above) in the absence and presence of thiosulfate and both dilution series were then incubated for 30 minutes at 37°C (5% CO_2_, humidified atmosphere). Media was then removed from the cells and competing ligand (in the presence or absence of thiosulfate) added to the wells immediately followed by [3H]prazosin (thus thiosulfate was present with the competing ligand for 30 minutes before addition to the cells, and then throughout the 2‐hour incubation with cells at 1 mmol/L).

[3H]prazosin concentrations were determined from taking the average of triplicate 50 µL samples of each [3H]prazosin concentration used and counted on a PerkinElmer Scintillation counter and were in the range from 0.22 to 1.40 nmol/L.

All experiments have been conducted in intact living mammalian cells expressing human α1A or α1B or α1D‐adrenoceptors. Unlike membrane‐binding studies, physiological levels of intracellular endogenous GTP will therefore always have been present. Although it should not make much difference for antagonists, the receptors (and therefore measurements taken) in this living system are therefore more akin to how drugs bind in people, than studies conducted in membrane preparations.

### 2.6 Data analysis

In all cases where a K_D_ value is stated, increasing concentrations of the competing ligand fully inhibited the specific binding of [3H]prazosin (unless otherwise annotated in the tables).

The following equation was then fitted to the data using Graphpad Prism 7 and the IC_50_ was then determined as the concentration required to inhibit 50% of the specific binding.

$$\%\text{specific binding}=100-\frac{\left(100\times [A]\right)}{\left(\left[A\right]+{\text{IC}}_{50}\right)}$$

where [A] is the concentration of the competing ligand and IC_50_ is the concentration at which half of the specific binding of [3H]prazosin has been inhibited.

From the IC_50_ value, the known concentration of [3H]prazosin and the known K_D_ for [3H]prazosin at each receptor, a K_D_ (concentration at which half the receptors are bound by the competing ligand) value was calculated using the Cheng–Prusoff equation:

$$K_{D}=\frac{{\text{IC}}_{50}}{1+\left([[3H]\text{prazosin}]/K_{D}\left[3H\right]\text{prazosin}\right)}$$

In some cases, the maximum concentration of competing ligand was not able to inhibit all of the specific binding. Where no inhibition of [3H]prazosin binding was seen, even with maximum concentration of competing ligand possible, “no binding” is given in the tables. Where the inhibition produced by the maximum concentration of the competing ligand was 50% or less, an IC_50_ could not be determined and thus a K_D_ value not calculated. This is shown in the tables as IC_50_ > top concentration used (ie, IC_50_ > 100 µmol/L means that 100 µmol/L inhibited some but less than 50% of the specific binding). In cases where the competing ligand caused a substantial (greater than 60%, but not 100%) inhibition of specific binding, an IC_50_ value was determined by extrapolating the curve to nonspecific levels and assuming that a greater concentration would have resulted in 100% inhibition. These values are given as apparent K_D_ values in the tables.

For some ligands, the inhibition of [3H]prazosin binding was best described by a two‐component curve, using the equation below:

$$\%\text{specific binding}=\frac{[A].N}{\left(\left[A\right]+{\text{IC}}_{50}1\right)}+\frac{\left[A].(100-N\right)}{\left(\left[A\right]+{\text{IC}}_{50}2\right)}$$

where [A] is the concentration of the competing ligand, IC_50_1 and IC_50_2 are the respective IC_50_ values for the two components and N is the percentage of the response occurring through the first component (IC_50_1). K_D_ values were calculated from IC_50_ values as above.

Selectivities are given as a ratio of the K_D_ values for the different receptors.

## 3 RESULTS

Saturation binding yielded a K_D_ value for [3H]prazosin of 0.71 nmol/L ± 0.07 (1552 ± 166 fmol/mg protein, n = 11) at the human α1A‐adrenoceptor, 0.87nM ± 0.11 (4350 ± 317 fmol/mg protein, n = 12) at the human α1B‐adrenoceptor, and 1.90 ± 0.31 nmol/L (417 ± 48 fmol/mg protein, n = 9) at the full‐length human α1D‐adrenoceptor. As the lower expression of the α1D‐receptor meant that a larger proportion of the experimental window was nonspecific binding, the affinity of prazosin was also determined by competing prazosin with [3H]prazosin. The log K_D_ values obtained were −9.07 ± 0.04 (=0.85 nmol/L, n = 9) at the α1A‐adrenoceptor, −8.74 ± 0.06 (=1.82 nmol/L, n = 8) at the α1B‐adrenoceptor, and −9.07 ± 0.23 (=0.85 nmol/L, n = 10) at the α1D‐ adrenoceptor. These values are all within twofold of the value obtained from saturation studies. The values from saturation studies were used for further K_D_ calculations. A lower receptor expression level for the full‐length α1D‐adrenoceptors is a common finding^15^, ^33^ and reports suggest truncation of the N‐terminus results in higher receptor expressions.^33^, ^34^, ^35^

**FIGURE 1:**
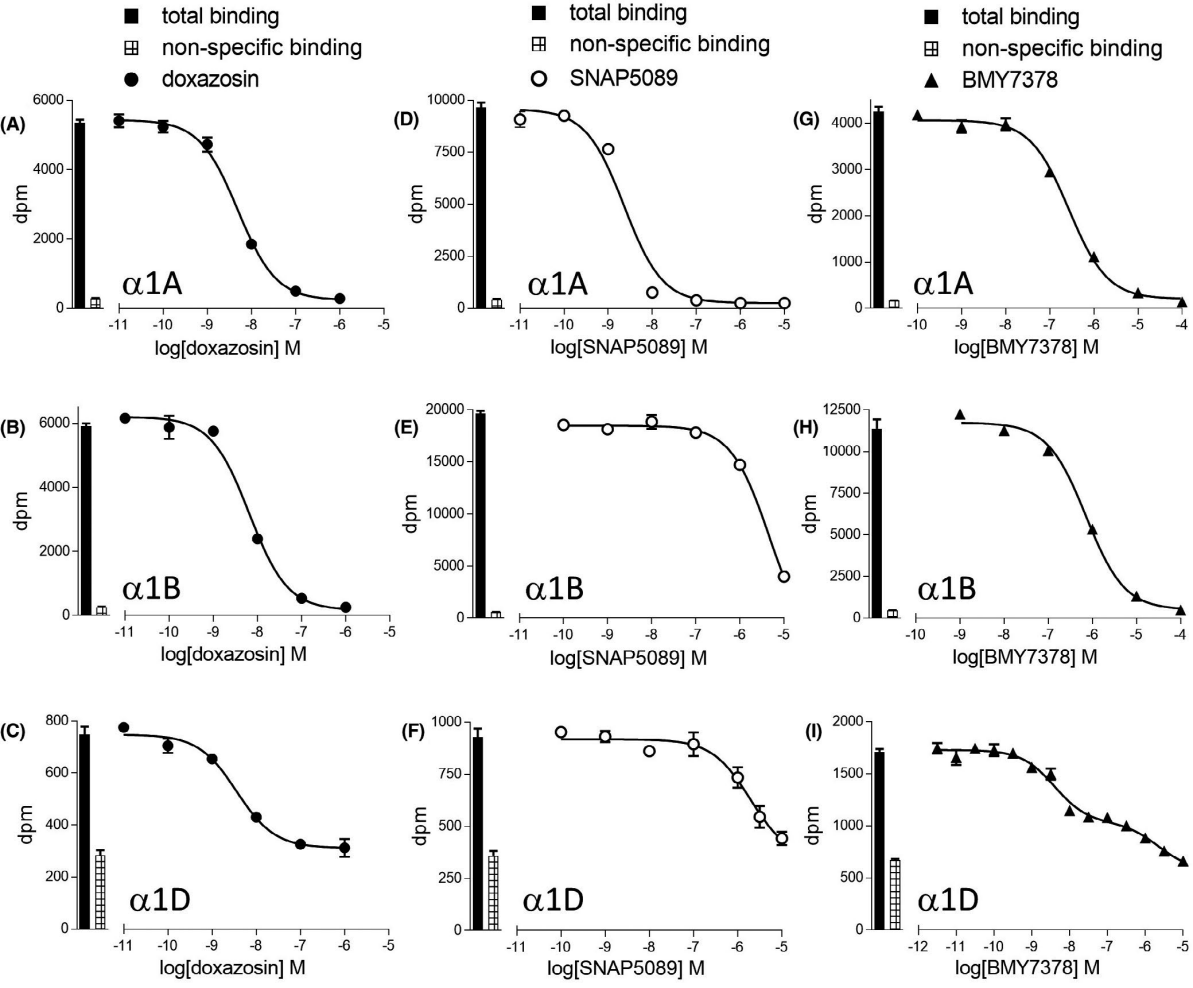
Inhibition of [3H]prazosin binding to whole cells by doxazosin (A–C), SNAP5089 (D–F) or BMY7378 (G–I) to CHO‐α1A cells (A, D, G), CHO‐α1B cells (B, E, H), or CHO‐α1D cells (C, F, I). Bars represent total [3H]prazosin binding and nonspecific binding was determined in the presence of 10 μmol/L tamsulosin (CHO‐α1A and CHO‐α1B) or 100 μmol/L tamsulosin (CHO‐α1D). The concentration of [3H]prazosin was (A) 0.31 nmol/L, (B) 0.31 nmol/L, (C) 0.70 nmol/L, (D) 0.68 nmol/L, (E) 0.68 nmol/L, (F) 0.60 nmol/L, (G) 0.24 nmol/L, (H) 0.42 nmol/L, and (I) 1.25 nmol/L. Data points are mean ± SE mean of triplicate determinations

Doxazosin, a commonly used α blocker in the treatment of hypertension, inhibited all three receptors with high affinity (log K_D_ −8.58, −8.46, and −8.33 at the α1A, α1B, and α1D‐adrenoceptor, respectively, Figure 1, Table 1). Of all the compounds studied, SNAP 5089 had the highest receptor selectivity, being over 1700‐fold selective for the α1A‐adrenoceptor (Figure 1, Table 1). No compound was found to have α1B‐adrenoceptor selectivity. The ability of BMY7378 to inhibit [3H]prazosin binding was best described by a two‐component curve with the high‐affinity component (log K_D_ −8.60 at the α1D‐adrenoceptor) giving it 98‐ and 234‐fold selectivity for the α1D‐adrenoceptor over the α1A and α1B‐adrenoceptors, respectively (Figure 1, Table 1). Several compounds had affinities of less than 0.25nM, including ligands with α1A selectivity (silodosin, RS100329, and tamsulosin), cyclazosin with slight α1D selectivity), and nonselective 3‐MPPI (Table 1).

**TABLE 1:** Log K_D_ values and selectivity ratios of α‐antagonists binding to the human α1A, α1B and α1D‐adrenoceptors. Values represent mean ± SE mean of n separate experiments. Selectivity ratios are also given where a ratio of 1 demonstrates no selectivity for a given receptor subtype over another. Thus, SNAP5089 has 1778 fold higher affinity for the α1A‐adrenoceptor than the α1B‐adrenoceptor. Compounds are arranged in order of α1A‐selectivity

Ligand	Log *K_D_* α1A	n	Log *K_D_* α1B	n	Log *K_D_* α1D	n	α1A	vs	α1B	α1A	vs	α1D	α1B	vs	α1D
α‐antagonists															
SNAP5089	−8.89 ± 0.03	6	−5.64 ± 0.04^app^	6	−5.65 ± 0.09^app^	5	1778			1738				1.0	
Silodosin	−9.61 ± 0.08	9	−6.50 ± 0.09	9	−6.94 ± 0.16	9	1288			468					2.8
RS100329	−9.60 ± 0.05	10	−6.67 ± 0.07	7	−7.63 ± 0.17	6	851			93					9.1
					−5.13 ± 0.15										
					70.5 ± 3.0% site 1										
Niguldipine	−9.24 ± 0.11	9	−6.33 ± 0.08	5	−5.92 ± 0.06	6	813			2089			2.6		
5‐methyl‐urapidil	−8.23 ± 0.05	5	−6.06 ± 0.04	5	−5.61 ± 0.07	5	148			417			2.8		
Lisuride	−7.94 ± 0.06	5	−6.07 ± 0.04	5	−6.93 ± 0.11	7	74			10					7.2
Benoxathian	−9.08 ± 0.05	6	−7.32 ± 0.03	6	−7.91 ± 0.10	7	58			15					3.9
					−5.62 ± 0.19										
					62.5 ± 3.4% Site 1										
Anisodamine	−5.21 ± 0.03	5	−3.45 ± 0.04^app^	5	−4.21 ± 0.05	5	58			10					5.8
RS17053	−8.33 ± 0.09	11	−6.61 ± 0.09	11	−6.84 ± 0.13	6	52			31					1.7
Urapidil	−7.21 ± 0.02	5	−5.50 ± 0.07	7	−6.37 ± 0.10	6	51			6.9					7.4
2‐MPMDQ	−9.06 ± 0.07	6	−7.37 ± 0.04	6	−9.01 ± 0.16	8	49			1.1					44
					−5.66 ± 0.29										
					64.0 ± 2.1% site1										
WB4104	−9.03 ± 0.04	10	−7.39 ± 0.05	7	−8.63 ± 0.11	9	44			2.5			17		
					−5.96 ± 0.09										
					59.6 ± 3.2% site 1										
Indoramin	−8.43 ± 0.07	5	−6.82 ± 0.04	5	−6.29 ± 0.07	5	41			138			3.4		
Phentolamine	−8.15 ± 0.08	8	−6.55 ± 0.05	5	−6.84 ± 0.11	6	40			20					1.9
					−4.64 ± 0.14										
					60.5 ± 3.8% site 1										
Tamsulosin	−9.67 ± 0.06	17	−8.12 ± 0.04	15	−9.18 ± 0.08	13	35			3.1					11
					−5.67 ± 0.15										
					54.6 ± 3.7% site 1										
Amitraz	−5.52 ± 0.05	5	Log IC_50_ > −4	6	−5.08 ± 0.05	5	>33			2.8					>12
Labetolol	−7.33 ± 0.04	7	−5.91 ± 0.03	7	−6.12 ± 0.07	6	26			16					1.6
Domperidone	−6.85 ± 0.12	6	−5.50 ± 0.05	5	−5.98 ± 0.05	5	22			7.4					3.0
Dibenamine	−7.91 ± 0.06	15	−6.57 ± 0.07	14	−7.37 ± 0.15	9	22			3.5					6.3
	−5.32 ± 0.08		−4.66 ± 0.06		−5.00 ± 0.14										
	83.0 ± 1.8% site 1		67.6 ± 2.6% site 1		47.8 ± 3.2% site 1										
Atipamezole	−5.99 ± 0.03	5	−4.68 ± 0.08	6	−5.33 ± 0.04	5	20			4.6					4.5
MK‐912	−6.76 ± 0.03	5	−5.46 ± 0.05	5	−7.30 ± 0.16	7	20					3.5			69
					−5.50 ± 0.25										
					61.2 ± 5.5% site 1										
2‐PMDQ	−8.19 ± 0.09	5	−6.95 ± 0.05	6	−8.42 ± 0.12	9	17					1.7			30
					−5.61 ± 0.12										
					57.6 ± 2.8% site 1										
BRL44408	−5.92 ± 0.09	9	−4.68 ± 0.07	9	−5.06 ± 0.05	5	17			7.2					2.4
ARC239	−9.35 ± 0.08	8	−8.15 ± 0.07	9	−8.74 ± 0.12	7	16			4.1					3.9
					−5.42 ± 0.21										
					60.5 ± 1.4% site 1										
Efaroxan	−5.47 ± 0.03	5	−4.27 ± 0.07	5	−4.97 ± 0.06	5	16			3.2					5.0
Ifenprodil	−7.66 ± 0.11	9	−6.49 ± 0.07	6	−8.12 ± 0.18	8	15			2.9					43
					−6.05 ± 0.13										
					48.8 ± 4.5% site 1										
Naftopidil	−7.97 ± 0.03	6	−6.82 ± 0.06	6	−7.06 ± 0.11	7	14			8.1					1.7
SKF86466	−6.06 ± 0.05	5	−4.93 ± 0.05	5	−5.16 ± 0.09	5	13			7.9					1.7
Sunepitron	−5.78 ± 0.06	5	−4.65 ± 0.06	5	−5.33 ± 0.23	6	13			2.8					4.8
RX821002	−6.51 ± 0.09	6	−5.46 ± 0.06	6	−5.31 ± 0.13	7	11			16			1.4		
3‐MPPI	−9.57 ± 0.06	6	−8.59 ± 0.03	6	−9.76 ± 0.15	7	9.5					1.5			15
					−6.93 ± 0.17										
					66.7 ± 3.4% site 1										
S32212	−5.90 ± 0.06	5	−4.92 ± 0.02^app^	5	−5.69 ± 0.13^app^	5	9.5			1.6					5.9
Promethazine	−7.00 ± 0.10	11	−6.06 ± 0.05	10	−5.75 ± 0.07	5	8.7			18			2.0		
AH11110A	−6.48 ± 0.03	5	−5.65 ± 0.09	5	−4.98 ± 0.06	5	6.8			32			4.7		
Yohimbine	−6.23 ± 0.03	5	−5.44 ± 0.05	5	−6.20 ± 0.08	8	6.2			1.1					5.8
Idazoxan	−5.67 ± 0.07	5	−4.88 ± 0.03	5	−5.23 ± 0.11	5	6.2			2.8					2.2
Bromocriptine	−8.73 ± 0.06	5	−7.96 ± 0.07	5	−7.31 ± 0.15^early plateau^	9	5.9			26			4.5		
Phenoxybenzamine	−8.45 ± 0.12	12	−7.69 ± 0.06	13	−8.43 ± 0.19	10	5.8			1.0					5.5
	−6.02 ± 0.08		−5.57 ± 0.06		−5.42 ± 0.08										
	77.7 ± 5.2% site 1		67.5 ± 2.5% site 1		39.1 ± 2.0% site 1										
RS79948	−5.75 ± 0.05	5	−4.99 ± 0.05	5	−6.07 ± 0.11	6	5.8					2.1			12
JP1302	−6.21 ± 0.04	5	−5.46 ± 0.02	5	−5.58 ± 0.09	5	5.6			4.3					1.3
Imiloxan	−4.60 ± 0.05	5	IC_50_ > −4	5	−5.02 ± 0.07	5	>4.0					2.6			>10
HEAT	−8.57 ± 0.06	5	−8.04 ± 0.04	5	−8.11 ± 0.18	8	3.4			2.9			1.2		
					−5.15 ± 0.26										
					64.1 ± 4.1% site 1										
Carvedilol	−−8.35 ± 0.06	12	−7.84 ± 0.06	6	−7.87 ± 0.12	7	3.2			3.0					1.1
A80426	−6.57 ± 0.05	5	−6.08 ± 0.02	5	−6.07 ± 0.07	5	3.1			3.1					1.0
Rec15‐2615	−8.26 ± 0.10	6	−7.79 ± 0.09	6	−7.89 ± 0.07	7	3.0			2.3					1.3
Spiroxatrine	−6.86 ± 0.06	5	−6.41 ± 0.06	5	−7.86 ± 0.13	6	2.8					10			28
					−5.84 ± 0.31										
					61.9 ± 4.0% site 1										
BMY7378	−6.61 ± 0.05	5	−6.23 ± 0.05	6	−8.60 ± 0.13	9	2.4					98			234
					−5.93 ± 0.37										
					57.7 ± 2.6% site 1										
Prazosin	−9.07 ± 0.04	9	−8.74 ± 0.06	8	−9.07 ± 0.23	10	2.1				1.0				2.1
Alfuzosin	−7.82 ± 0.11	8	−7.56 ± 0.08	6	−7.66 ± 0.11	6	1.8			1.4					1.3
Cyclazosin	−8.89 ± 0.06	7	−8.68 ± 0.08	5	−9.87 ± 0.06	7	1.6					9.5			15
					−7.44 ± 0.10										
					56.8 ± 3.4% site 1										
Doxazosin	−8.58 ± 0.09	6	−8.46 ± 0.05	8	−8.33 ± 0.13	11	1.3			1.8			1.3		
Terazosin	−7.93 ± 0.05	6	−7.95 ± 0.05	6	−7.71 ± 0.13	7		1.0		1.7			1.7		
β‐blockers															
Carazolol	−6.57 ± 0.03	5	−4.68 ± 0.05	6	−5.04 ± 0.08^app^	5	78			34					2.3
SDZ21009	−5.24 ± 0.07	6	Log IC_50_ > −4	6	−5.09 ± 0.18	6	>17			1.4					>12
CGP12177	−5.14 ± 0.05	6	Log IC_50_ > −4	5	−4.20 ± 0.11	5	>14			8.7					>1.6
Bucindolol	−7.57 ± 0.07	5	−6.46 ± 0.04	5	−6.45 ± 0.09	5	13			13					1.0
ICI118551	−5.23 ± 0.03	5	−4.20 ± 0.06^app^	5	−4.96 ± 0.03	5	11			1.9					5.8
CGP20712A	−4.93 ± 0.10	5	Log IC_50_ > −4	5	−4.96 ± 0.07^app^	5	>8.5					1.1			>9.1
Propranolol	−4.91 ± 0.02	6	−3.98 ± 0.04^app^	6	−4.89 ± 0.09	5	8.5				1.0				8.1
Cyanopindolol	−5.59 ± 0.05	8	−4.91 ± 0.09	7	−5.40 ± 0.07	5	4.8			1.5					3.1

^app^The maximum concentration of competing ligand inhibited most but not all of specific binding (as in Figure 1E). An IC_50_ was determined by extrapolating the curve assuming that all specific binding would be inhibited if a higher concentration of competing ligand were possible. Thus an app *K_D_* was calculated.

^early plateau^Bromocriptine did not fully inhibit specific binding at the α1D‐adrenoceptor. The inhibition curve reached a plateau of maximal inhibition of binding at 71.0% ± 3.4% inhibition of specific binding (n = 9).

app, apparent.

**FIGURE 2:**
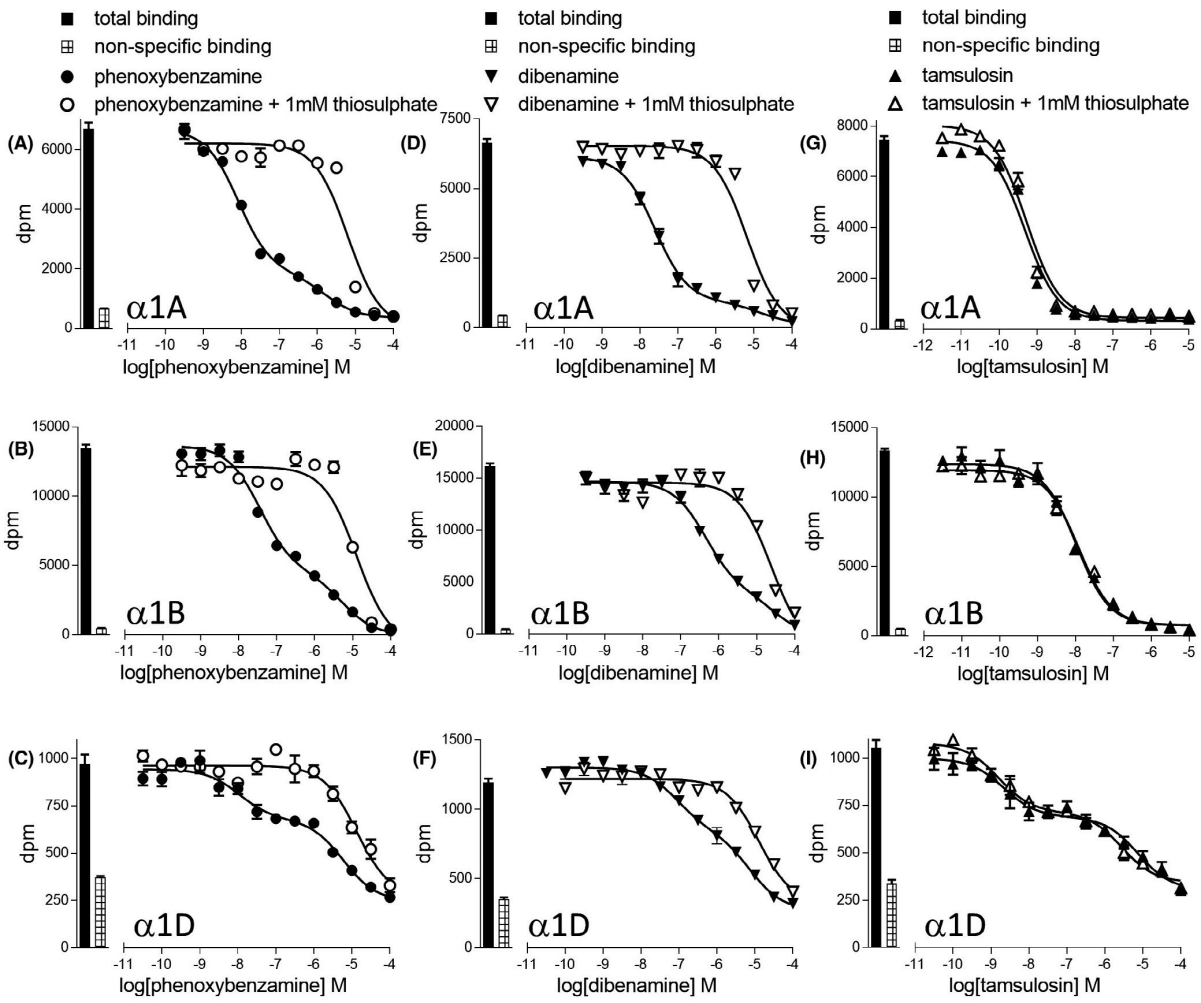
Inhibition of [3H]prazosin binding to whole cells by phenoxybenzamine (A–C), dibenamine (D–F) or tamsulosin (G–I) to CHO‐α1A cells (A, D, G), CHO‐α1B cells (B, E, H), or CHO‐α1D cells (C, F, I). Bars represent total [3H]prazosin binding and nonspecific binding was determined in the presence of 10 μmol/L tamsulosin (CHO‐α1A and CHO‐α1B) or 100 μmol/L tamsulosin (CHO‐α1D). The concentration of [3H]prazosin was (A) 0.48 nmol/L, (B) 0.48 nmol/L, (C) 0.86 nmol/L, (D) 0.58 nmol/L, (E) 0.56 nmol/L, (F) 1.49 nmol/L, (G) 0.56 nmol/L, (H) 0.58 nmol/L, and (I) 1.49 nmol/L. Data points are mean ± SE mean of triplicate determinations

Two compounds were best described by a two‐component‐binding inhibition curve at all three receptors—phenoxybenzamine and dibenamine (Figure 2, Table 1). Both of these are *N,N*‐disubstituted‐2‐chloroethylamines. Preincubation of phenoxybenzamine and dibenamine with sodium thiosulfate before addition to the cells yielded a single‐component‐binding inhibition (Figure 2, Table 2), whereby the high‐affinity‐binding component of the parent curve had been abolished. Sodium thiosulfate had no effect on the binding of tamsulosin (Figure 2, Table 2). At the α1D‐adrenoceptor, several other ligands were best described by a two‐component‐binding inhibition curve. Just as with tamsulosin (Figure 2), preincubation with sodium thiosulfate had no effect on either component of any of these other two‐component ligands.

**TABLE 2:** Log *K_D_* values of phenoxybenzamine, dibenamine and tamsulosin binding to the human α1A, α1B and α1D‐adrenoceptors obtained in the absence and presence of 1 mmol/L sodium thiosulphate (Figure 2). Values represent mean ± SE mean of n separate experiments

		Control			+1 mmol/L sodium thiosulphate			
	Log *K_D_* site 1	Log *K_D_* site 2	% site 1	n	Log *K_D_* site 1	Log *K_D_* site 2	% site 1	n
CHO‐α1A								
Phenoxybenzamine	−8.45 ± 0.12	−6.02 ± 0.08	77.7 ± 5.2	12	−5.43 ± 0.07			7
Dibenamine	−7.91 ± 0.06	−5.32 ± 0.08	83.0 ± 1.8	15	−5.16 ± 0.10			7
Tamsulosin	−9.67 ± 0.06			17	−9.75 ± 0.16			7
CHO‐α1B								
Phenoxybenzamine	−7.69 ± 0.06	−5.57 ± 0.06	67.5 ± 2.5	13	−5.18 ± 0.05			6
Dibenamine	−6.57 ± 0.07	−4.66 ± 0.06	67.6 ± 2.6	14	−4.85 ± 0.05			6
Tamsulosin	−8.12 ± 0.04			15	−8.13 ± 0.08			6
CHO‐α1D								
Phenoxybenzamine	−8.43 ± 0.19	−5.42 ± 0.08	39.1 ± 2.0	10	−4.93 ± 0.10			5
Dibenamine	−7.37 ± 0.15	−5.00 ± 0.14	47.8 ± 3.2	9	−4.74 ± 0.09			5
Tamsulosin	−9.18 ± 0.08	−5.67 ± 0.15	54.6 ± 3.7	13	−9.11 ± 0.12	−5.60 ± 0.08	44.0 ± 2.8	7

**FIGURE 3:**
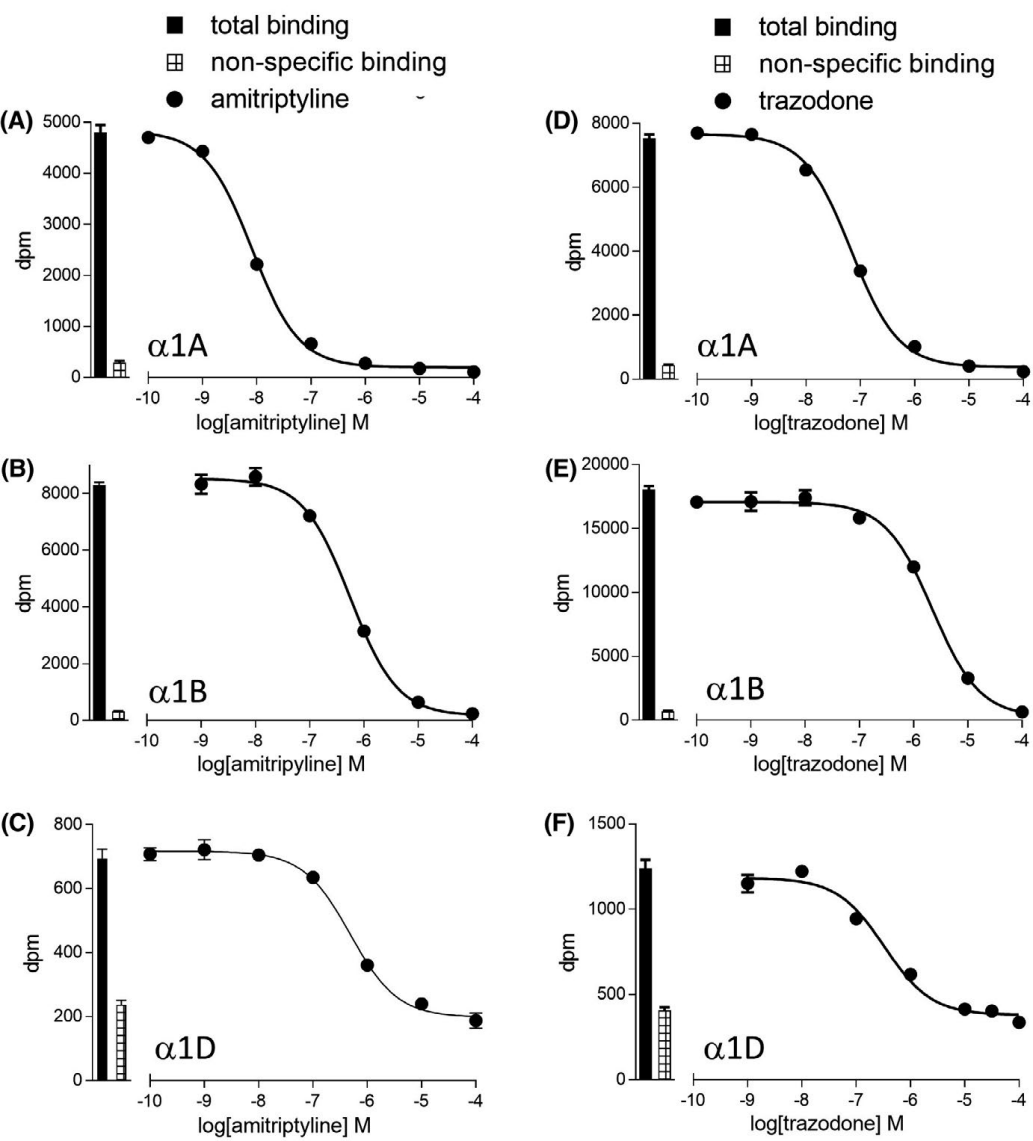
Inhibition of [3H]prazosin binding to whole cells by two commonly prescribed antidepressants amtriptyline (A–C) or trazodone (D–F) to CHO‐α1A cells (A, D), CHO‐α1B cells (B, E), or CHO‐α1D cells (C, F). Bars represent total [3H]prazosin binding and nonspecific binding was determined in the presence of 10 μmol/L tamsulosin (CHO‐α1A and CHO‐α1B) or 100 μmol/L tamsulosin (CHO‐α1D). The concentration of [3H]prazosin was a) 0.39 nmol/L, (B) 0.45 nmol/L, (C) 0.57 nmol/L, (D) 0.66 nmol/L, (E) 0.45 nmol/L, and (F) 0.66 nmol/L. Data points are mean ± SE mean of triplicate determinations

**FIGURE 4:**
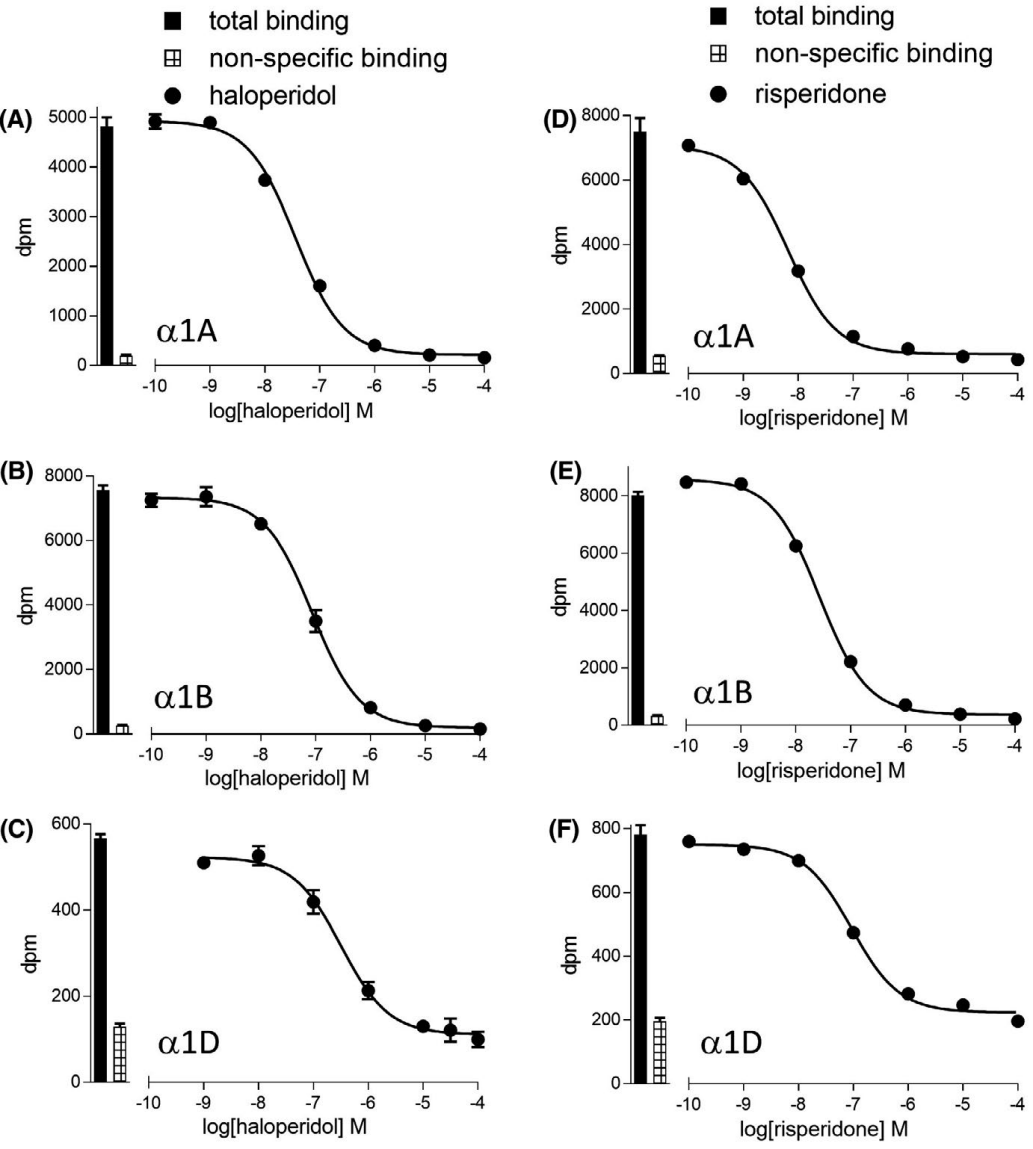
Inhibition of [3H]prazosin binding to whole cells by two commonly prescribed antipsychotics haloperidol (A–C) or risperidone (D–F) to CHO‐α1A cells (A, D), CHO‐α1B cells (B, E) or CHO‐α1D cells (C, F). Bars represent total [3H]prazosin binding and nonspecific binding was determined in the presence of 10 μmol/L tamsulosin (CHO‐α1A and CHO‐α1B) or 100 μmol/L tamsulosin (CHO‐α1D). The concentration of [3H]prazosin was (A) 0.39 nmol/L, (B) 0.39 nmol/L, (C) 0.53 nmol/L, (D) 0.82 nmol/L, (E) 0.45 nmol/L, and (F) 0.66 nmol/L. Data points are mean ± SE mean of triplicate determinations

The affinity of several antidepressants and antipsychotics was then examined. Several of these were found to have high α1‐adrenoceptor affinity (Figures 3 and 4, Table 3 and 4). Risperidone (previously suggested to have α1B selectivity,^4^, ^36^ had slight α1A selectivity, in keeping with the findings of^37^. There have also been discrepancies in the affinity of olanzapine: Richelson and Souder^27^ found it to have high affinity (44nM for α1‐adrenoceptor) and Nourain et al,^29^ had conflicting data with low rat α1‐adreoceptor affinity, but significant hypotension in rats. However, here, olanzepine had low affinity, in keeping with^38^ and the findings of^39^ where olanzapine was described as having low postural hypotension potential. WB4104, was also initially thought to have α1B selectivity,^40^ however, it had higher and equal affinity for α1A and α1D‐adrenoceptors (in keeping with^41^, ^42^).

**TABLE 3:** Log K_D_ values and selectivity ratios of antidepressants binding to the human α1A, α1B and α1D‐adrenoceptors. Values represent mean ± SE mean of n separate experiments. Selectivity ratios are also given where a ratio of 1 demonstrates no selectivity for a given receptor subtype over another. Thus, amitriptyline has 93 fold higher affinity for the α1A‐adrenoceptor than the α1B‐adrenoceptor. Compounds are arranged in order of α1A‐selectivity

Ligand	Log *K_D_* α1A	n	Log *K_D_* α1B	n	Log *K_D_* α1D	n	α1A	vs	α1B	α1A	vs	α1D	α1B	vs	α1D
Noradrenaline and serotonin reuptake inhibitors															
Tricyclic antidepressants															
Amitriptyline	−8.19 ± 0.02	9	−6.22 ± 0.05	9	−6.25 ± 0.05	5	93			87					1.1
Dosulepin	−7.11 ± 0.04	5	−5.28 ± 0.11	7	−5.58 ± 0.05	5	68			34					2.0
Clomipramine	−8.12 ± 0.10	9	−6.34 ± 0.07	9	−6.15 ± 0.09	5	60			93			1.5		
Imipramine	−7.47 ± 0.04	6	−5.76 ± 0.05	6	−5.89 ± 0.05	5	51			38					1.3
Norclomipramine	−7.52 ± 0.08	11	−5.84 ± 0.04	12	−5.84 ± 0.06	5	48			48				1.0	
Nortriptyline	−7.74 ± 0.03	6	−6.07 ± 0.07	5	−5.81 ± 0.05	5	47			85			1.8		
Doxepin	−7.74 ± 0.04	5	−6.18 ± 0.03	5	−6.27 ± 0.11	6	36			30					1.2
Desipramine	−7.07 ± 0.05	6	−5.57 ± 0.05	5	−5.46 ± 0.07	5	32			41			1.3		
Lofepramine	−6.94 ± 0.06	6	−5.44 ± 0.07	6	−5.37 ± 0.04	5	32			37			1.2		
Trimipramine	−7.37 ± 0.08	5	−6.10 ± 0.06	5	−5.99 ± 0.05	5	19			24			1.3		
Protriptyline	−6.67 ± 0.03	5	−5.57 ± 0.02	5	−5.46 ± 0.12	5	13			16			1.3		
Tetracyclic antidepressants															
Mirtazepine	−6.36 ± 0.02	5	−5.36 ± 0.03	5	−5.94 ± 0.05	5	10			2.6					3.8
Other noradrenaline and serotonin reuptake inhibitors															
Duloxetine	−5.65 ± 0.05	5	−4.71 ± 0.03^app^	5	−5.58 ± 0.12	7	8.7			1.2					7.4
Venlafaxime	−3.69 ± 0.02^app^	5	No binding to 1 mmol/L	5	−4.38 ± 0.16	7	>4.9					4.9			>24
Noradrenaline reuptake inhibitors															
Reboxetine	−4.91 ± 0.08	5	Log IC_50_ > −4	5	−4.67 ± 0.12	5	>8.1			1.7					>4.7
Selective serotonin reuptake inhibitors (SSRI)															
Fluvoxamine	−6.10 ± 0.03	5	Log IC_50_ > −4	5	−4.97 ± 0.03	5	>126			14					>9.3
Citalopram	−5.95 ± 0.06	4	Log IC_50_ > −4	4	−4.91 ± 0.11	5	>89			11					>8.1
Fluoxetine	−5.45 ± 0.04	5	−4.41 ± 0.06	5	−4.90 ± 0.13	5	11			3.5					3.1
Paroxetine	−5.59 ± 0.09	5	Log IC_50_ > −5	5	−5.63 ± 0.13	5	>3.9					1.1			>4.3
Sertraline	−5.72 ± 0.04	5	−5.45 ± 0.05	5	−5.61 ± 0.04	5	1.9			1.3					1.4
Serotonin reuptake inhibitors															
Vortioxetine	−6.32 ± 0.05	5	−5.42 ± 0.02	5	−5.43 ± 0.08	5	7.9			7.8				1.0	
Trazodone	−7.33 ± 0.04	6	−6.56 ± 0.07	6	−6.38 ± 0.15	7	5.9			8.9			1.5		
Metalonin agonist															
Agomelatine	−4.57 ± 0.11^app^	5	No binding to 100 µmol/L	5	IC_50_ > −4.5	6	>3.7			1.1			>3.2		

^app^The maximum concentration of competing ligand inhibited most but not all of specific binding (as in Figure 1E). An IC_50_ was determined by extrapolating the curve assuming that all specific binding would be inhibited if a higher concentration of competing ligand were possible. Thus an app *K_D_* was calculated.

app, apparent.

**TABLE 4:** Log K_D_ values and selectivity ratios of antipsychotics binding to the human α1A, α1B and α1D‐adrenoceptors. Values represent mean ± SE mean of n separate experiments. Selectivity ratios are also given where a ratio of 1 demonstrates no selectivity for a given receptor subtype over another. Thus, chlorpromazine has 13 fold high affinity for the α1A‐adrenoceptor than the α1B‐adrenoceptor. Compounds are arranged in order of α1A‐selectivity

Ligand	Log *K_D_* α1A	n	Log *K_D_* α1B	n	Log *K_D_* α1D	n	α1A	vs	α1B	α1A	vs	α1D	α1B	vs	α1D
First generation antipsychotics															
Sulpiride	−4.50 ± 0.07	5	IC_50_ > 3	5	−3.66 ± 0.09^app^	5	>32			6.9					>4.6
Chlorpromazine	−8.94 ± 0.06	5	−7.84 ± 0.05	5	−8.00 ± 0.08	6	13			8.7					1.4
					−5.91 ± 0.20										
					56.0 ± 5.0% site 1										
Flupenthixol	−8.35 ± 0.05	5	−7.47 ± 0.07	5	−6.96 ± 0.12	7	7.6			25			3.2		
Trifluoperazine	−7.75 ± 0.03	5	−6.88 ± 0.07	5	−6.36 ± 0.08	5	7.4			25			3.3		
Prochlorperazine	−7.61 ± 0.12	4	−6.88 ± 0.06	5	−6.53 ± 0.13	8	5.4			12			2.2		
Perphenazine	−8.15 ± 0.09	5	−7.43 ± 0.08	5	−7.86 ± 0.10	5	5.2			1.9					2.7
					−6.03 ± 0.19										
					53.3 ± 4.1% site 1										
Pimozide	−7.44 ± 0.16	5	−6.79 ± 0.05	5	−5.95 ± 0.08	6	4.5			31			6.9		
Haloperidol	−7.70 ± 0.03	5	−7.21 ± 0.07	6	−6.42 ± 0.06	5	3.1			19			6.2		
Second generation antipsychotics															
Amisulpiride	−5.05 ± 0.04	5	No binding to 100 µmol/L	5	−4.55 ± 0.08^app^	5	11			3.2					3.5
Ziprasidone	−8.73 ± 0.05	7	−7.70 ± 0.07	8	−7.20 ± 0.09	5	11			34			3.2		
Paliperidone	−8.36 ± 0.09	5	−7.36 ± 0.08	5	−7.47 ± 0.10	6	10			7.8					1.3
					−5.57 ± 0.21										
					57.6 ± 4.9% site 1										
Sertindole	−9.27 ± 0.09	9	−8.28 ± 0.11	8	−6.93 ± 0.12	8	9.8			219			22		
Risperidone	−8.74 ± 0.06	7	−7.77 ± 0.05	7	−7.14 ± 0.07	6	9.3			40			4.3		
Clozapine	−8.27 ± 0.04	5	−7.39 ± 0.07	5	−6.41 ± 0.05	5	7.6			72			9.5		
Quetiapine	−7.89 ± 0.10	5	−7.21 ± 0.04	5	−6.48 ± 0.10	5	4.8			26			5.4		
Lurasidone	−7.80 ± 0.11	5	−7.17 ± 0.09	5	−8.19 ± 0.10	7	4.3			2.5					10
					−5.92 ± 0.06										
					24.8 ± 3.1% site 1										
Aripiprazole	−7.32 ± 0.07	6	−6.69 ± 0.03	6	−6.15 ± 0.11	5	4.3			15			3.5		
Olanzapine	−6.61 ± 0.11	7	−6.00 ± 0.10	10	−5.86 ± 0.06	5	4.1			5.6			1.4		

^app^The maximum concentration of competing ligand inhibited most but not all of specific binding (as in Figure 1E). An IC_50_ was determined by extrapolating the curve assuming that all specific binding would be inhibited if a higher concentration of competing ligand were possible. Thus an app *K_D_* was calculated.

app, apparent.

Tables combing all of these ligands are presented in Supplementary Data. Table S1 has the ligands arranged in alphabetical order (together with their suppliers and individual ligand codes). Table S2 has the ligands organized in order of α1A affinity.

## 4 DISCUSSION

Dibenamine, phentolamine, and phenoxybenzamine were the first clinical α blockers^43^ and phenoxybenzamine is still used in the management of pheochromocytoma, particularly during surgery where catastrophic catecholamine release can cause hypertensive crises and arrhythmias.^7^ Both phenoxybenzamine and dibenamine are *N,N*‐disubstituted‐2‐chloroethylamines containing a nitrogen mustard group. Both compounds were best described by a two‐component‐binding inhibition curve at all three α1‐adrenoceptors (Figure 2, Table 1). In aqueous solution at physiological pH, the nitrogen mustard group cyclizes to form ethyleniminium ions.^44^ These highly reactive, unstable ions are pharmacologically active and covalently bind to a cysteine in transmembrane 3 of the α adrenoceptors, giving these compounds their “irreversible” properties.^43^ Phenoxybenzamine has a longer duration of action in clinical studies than phentolamine ^7^ and hence its continued use in pheochromocytoma (although similar outcomes have been reported with doxazosin, terazosin, and prazosin,.^45^, ^46^, ^47^ Sodium thiosulfate also rapidly reacts with the ethyleniminium ions thus prevents them from interacting with α adrenoceptors.^44^ Pretreatment with intravenous sodium thiosulfate prevented dibenamine binding to α adrenoceptors (in cats,^48^ and pretreatment with sodium thiosulfate prevented the harmful interactions of the chemical weapon mustard gas in humans. Here, preincubation of phenoxybenzamine or dibenamine with sodium thiosulfate yielded single‐component‐binding inhibition curves (Figure 2, Table 2). Abolishment of the high‐affinity‐binding component suggests that it was due to specific α1‐adrenoceptor interaction. The K_D_ values of the low‐affinity components were very similar to those obtained in the presence of thiosulfate, suggesting that this component is a non‐orthosteric site or non‐receptor‐mediated effect.

Several other ligands were found to have a [3H]prazosin inhibition best described a two‐component curve at the α1D‐adrenoceptor, including tamsulosin (and hence why 100 μM was used to define nonspecific binding in CHO‐α1D cells, rather than 10 μM used in α1A and α1B cells), and the only α1D‐selective ligand, BMY7378. As expected for these nonmustard compounds, preincubation with sodium thiosulfate had no effect on binding. The reason for the second component is therefore unknown. Affinity (K_D_ value) obtained for the high‐affinity component the α1D‐adrenoceptor has been used to determine receptor selectivity.

α1‐adrenoceptor antagonists (α blockers, especially doxazosin) have been used for hypertension for decades. Doxazosin had high affinity for all three subtypes, similar to previous [3H]prazosin‐binding studies.^41^ Terazosin and prazosin were also nonselective ligands (as in^41^) as was phentolamine. Indoramin, (licensed for hypertension), had an α1A selectivity of 40‐fold (similar to^50^). Of the α blockers that are used for the treatment of BPH in the UK, alfuzosin was nonselective, whereas tamsulosin with its α1A vs α1B selectivity of 35‐fold, was equipotent at α1A and α1D receptors (as in 15, 16, 51). Thus, drugs used for hypertension and BPH include nonselective α1 blockers and those with up to 40‐fold α1A selectivity. It would therefore be expected that drugs like tamsulosin and alfuzosin, licensed for BPH, are likely to have as much of an effect on blood pressure as α blockers intentionally prescribed for hypertension. Several other high‐affinity non/poorly selective ligands were also identified that have higher affinity than doxazosin, for example, cyclazosin, 3‐MPPI, and ARC239.

Carvedilol (commonly used in heart failure) is considered a dual α/β blocker. Carvedilol was nonselective, with high affinity at all three α1‐adrenoceptors, however the α1A affinity (log K_D_ of −8.35) was still 10‐fold less than that for the β2 adrenoceptor.^52^ Labetolol (used in hypertension particularly in pregnancy, and intravenously in hypertensive emergencies), is also considered a dual α/β blocker. Labetolol has lower affinity than carvedilol for all β^52^ and α1‐adrenoceptors (log K_D_ −7.33 at α1A), but very poor affinity for the α1B and α1D‐adrenoceptors. Labetolol should be considered a β1/β2/α1A blocker rather than dual pan α1 and β blocker. Given these dual α/β ligands, the affinity of a few β blockers with very high β‐adrenoceptor affinity were examined (Table 1). With the exception of bucindolol, the affinity was poor at all three α1‐adrenoceptors, confirming their β selectivity. Although the affinity of bucindolol was reasonably high (log K_D_ at α1A −7.57), this is 54‐fold and 263‐fold lower than that for the human β1 and β2 adrenoceptor, respectively.^53^

The most selective ligand detected here was SNAP5089, with 1700‐fold selectivity for the α1A over the α1B or α1D‐adrenoceptors. Other α1A‐selective ligands were silodosin, RS100329, and niguldipine (in keeping with^50^, ^54^). As well as tamsulosin, several ligands had higher affinity for the α1A and α1D receptors than the α1B—for example, 2‐MPMDQ, MK‐912, 2‐PMDQ, and ifenprodil. BMY7378 was the only compound with substantial α1D selectivity (the 100‐ to 200‐fold selectivity is similar to^6^, ^20^, ^41^, ^50^, ^55^). No α1B‐selective ligand was identified. To pharmacologically infer the presence of α1B‐adrenoceptors in cells or tissues, several different compounds with different patterns of selectivity would be required, for example, SNAP5089, doxazosin, 2‐MPMDQ, and BMY7378.

Several tricyclic antidepressants (TCA) had significant affinity for the α1‐adrenoceptors. Amitriptyline, clomipramine, doxepin, and nortriptyline have similar α1‐adrenoceptor affinities and selectivities to α blockers prescribed for hypertension or BPH. Thus patients taking these TCAs should be considered to be α blocked and are at risk from postural hypotension (as in ^23^). Furthermore, the addition of an α blocker for concomitant hypertension or BPH may not have any additional clinical benefit and may actually cause significant postural problems. Other TCA had lower affinity, for example, protriptyline and lofepramine and would therefore be expected to have less effect on blood pressure. The selective serotonin reuptake inhibitors (SSRIs) had very poor affinity for any of the α adrenoceptors and are therefore less likely to have significant α1‐mediated hypotension.

Several antipsychotics (including first‐generation chlorpromazine and flupenthixol and second‐generation sertindole, risperidone, and clozapine) had high α1‐adrenoceptor affinity. The very high affinity of sertindole (and 300‐fold selectivity for α1A over α1D‐adrenoceptors) was similar to previous reports.^6^, ^29^ The degree of α1A‐adrenoceptor affinity observed here correlates well with the rankings for observations in rats.^29^ The high α1A affinity of sertindole, risperidone, and ziprasidone (log K_D_ −9.3 to −8.7) is similar to studies,^38^ including in brain tissue,^27^ and similar to (or even higher than) that for many drugs used to treat hypertension. The high rate of postural hypotension observed with these drugs^31^, ^32^ is therefore not surprising. A similar hypotensive effect would be expected with other antipsychotics α1 affinities equal or greater than that for α1 blockers used for hypertension, for example, chlorpromazine, flupenthixol, perphenazine, paliperidone, quetiapine, and lurasidone. Aripiprazole had lower α1 affinity (in keeping with^56^ and indeed has a relative lack of reported postural hypotension in clinical studies^57^). However, sulpiride and amisulpiride would be expected to have even less hypotensive effect.

Thus, the high α1 affinity and selectivity profile of many antipsychotics is comparable to the α1 blockers intentionally prescribed for hypertension. Equivalent reductions in blood pressure are a likely very common side effect. These drugs are used to manage schizophrenia where their effect on blood pressure in agitated patients is less likely to be an issue. However, antipsychotics are also widely used to manage delirium in sick patients including the intensive care unit^58^, ^59^ and in palliative case^60^, ^61^ even though recent studies have questioned their effectiveness.^62^ Delirium is common in older unwell patients who are more likely to be suffering from conditions with lower blood pressure such as sepsis. In these cases, the choice of antipsychotic may well be important in order not to exacerbate already low or labile blood pressures. This study suggests that sulpiride, amisulpiride, ariprazole, and olazepine should have the least effect on blood pressure.

In conclusion, there are several highly α1A‐selective antagonists (eg, SNAP5089), and one α1D‐selective antagonist (BMY7378), however no α1B‐selective ligand has been identified. The drugs used for hypertension and BPH have a very similar pharmacological profile in terms of α1‐adrenoceptor subtype affinity and selectivity. Several antidepressants and antipsychotics have high α1‐adrenoceptor affinities, similar to, or even greater than, those seen for α blockers prescribed for hypertension and BPH. The addition of further α blockers for the management of hypertension or BPH in these patients may not be beneficial. The excellent correlation between the affinity values determined from this cell studies with the affinities measured in blood vessels, brain tissue, and whole animals (including humans) means that many, but not all antipsychotics and antidepressant may cause significant peripheral α‐adrenoceptor blockade and associated hypotension. Finally, awareness of the α‐blocking potential of certain, but not all antipsychotics may affect the choice of drug used for the management of delirium in the intensive care unit where additional α blockade and blood pressure lowering in a sick patient may be detrimental.

## DISCLOSURE

JGB has been on the Scientific Advisory Board for CuraSen Therapeutics since 2019.

## AUTHORS CONTRIBUTIONS

JGB designed the research study. ASP contributed the α1D DNA and aided discussions. RGWP and JGB performed the research. JGB analyzed the data. JGB and ASP wrote the paper.

## ETHICAL STATEMENT

No animals, human tissue, human volunteers, or patients were used in this study.

## DATA SHARING

Further information and requests for data and reagents should be directed to and will be fulfilled by the corresponding author, Jillian Baker. Please contact jillian.baker@nottingham.ac.uk.

### Open Research Badges

This article has earned an Open Data badge for making publicly available the digitally‐shareable data necessary to reproduce the reported results.

## Supporting information

Table S1‐S2
