# Supplementary material for: The affinity and selectivity of α‐adrenoceptor antagonists, antidepressants, and antipsychotics for the human α1A, α1B, and α1D‐adrenoceptors
Source: Pharmacol Res Perspect. 2020 Jul 1;8(4):e00602. doi: 10.1002/prp2.602 (PMC7327383; doi:10.1002/prp2.602)
Supplement: Supplementary file 1 — Table S1‐S2 [file PRP2-8-e00602-s001.docx]

**Supplementary data**

**Supplementary Data Table 1 – alphabetical order**

Alphabetical order for log K_D_ values of α-antagonists obtained from ^3^H-prazosin whole cell binding to the human α1A, α1B and α1D-adrenoceptors stably expressed in CHO cells. Ligand suppliers and their catalogue numbers are also given. Values represent mean ± s.e.mean of n separate experiments. Selectivity ratios are also given where a ratio of 1 demonstrates no selectivity for a given receptor subtype over another.

|  |  |  | Log K_D_ values determined from ^3^H-prazosin whole cell binding | | | | | |  | selectivities | | |
| --- | --- | --- | --- | --- | --- | --- | --- | --- | --- | --- | --- | --- |
| ligand | supplier | Catalogue  number | α1A | n | α1B | n | α1D | n |  | α1A vs α1B | α1A vs 1D | α1B vs α1D |
| A80426 | Tocris | 2341 | -6.57 ± 0.05 | 5 | -6.08 ± 0.02 | 5 | -6.07 ± 0.07 | 5 |  | 3.1 | 3.1 | 1.0 |
| agomelatine | sellakchem | S1243 | -4.57 ± 0.11^apparent^ | 5 | No binding to 100µM | 5 | Log IC_50_ >-4.5 | 6 |  | >3.7 | 1.1 | >3.2 |
| AH11110A | Sigma | A3477 | -6.48 ± 0.03 | 5 | -5.65 ± 0.09 | 5 | -4.98 ± 0.06 | 5 |  | 6.8 | 32 | 4.7 |
| alfuzosin | Sellakchem | S1409 | -7.82 ± 0.11 | 8 | -7.56 ± 0.08 | 6 | -7.66 ± 0.11 | 6 |  | 1.8 | 1.4 | 1.3 |
| amisulpiride | Sellakchem | S1280 | -5.05 ± 0.04 | 5 | No binding to 100µM | 5 | -4.55 ± 0.08 ^apparent^ | 5 |  | 11 | 3.2 | 3.5 |
| amitraz | Sigma | 45323 | -5.52 ± 0.05 | 5 | Log IC_50_>-4 | 6 | -5.08 ± 0.05 | 5 |  | >33 | 2.8 | >12 |
| amitriptyline | Sigma | A8404 | -8.19 ± 0.02 | 9 | -6.22 ± 0.05 | 9 | -6.25 ± 0.05 | 5 |  | 93 | 87 | 1.1 |
| anisodamine | Sigma | SML0252 | -5.21 ± 0.03 | 5 | -3.45 ± 0.04^apparent^ | 5 | -4.21 ± 0.05 | 5 |  | 58 | 10 | 5.8 |
| ARC239 | Sigma | A5736 | -9.35 ± 0.08 | 8 | -8.15 ± 0.07 | 9 | -8.74 ± 0.12  -5.42 ± 0.21  60.5 ± 1.4% site 1 | 7 |  | 16 | 4.1 | 3.9 |
| aripiprazole | Tocris | 5584 | -7.32 ± 0.07 | 6 | -6.69 ± 0.03 | 6 | -6.15 ± 0.11 | 5 |  | 4.3 | 15 | 3.5 |
| atipamezole | Sigma | A9611 | -5.99 ± 0.03 | 5 | -4.68 ± 0.08 | 6 | -5.33 ± 0.04 | 5 |  | 20 | 4.6 | 4.5 |
| benoxathian | Sigma | B016 | -9.08 ± 0.05 | 6 | -7.32 ± 0.03 | 6 | -7.91 ± 0.10  -5.62 ± 0.19  62.5 ± 3.4% site 1 | 7 |  | 58 | 15 | 3.9 |
| BRL44408 | Sigma | B4559 | -5.92 ± 0.09 | 9 | -4.68 ± 0.07 | 9 | -5.06 ± 0.05 | 5 |  | 17 | 7.2 | 2.4 |
| bromocriptine | Sigma | O427 | -8.73 ± 0.06 | 5 | -7.96 ± 0.07 | 5 | -7.31 ± 0.15  ^early plateau^ | 9 |  | 5.9 | 26 | 4.5 |
| bucindolol | Tocris | 2658 | -7.57 ± 0.07 | 5 | -6.46 ± 0.04 | 5 | -6.45 ± 0.09 | 5 |  | 13 | 13.2 | 1.0 |
| BMY7378 | Sellakchem | S2691 | -6.61 ± 0.05 | 5 | -6.23 ± 0.05 | 6 | -8.60 ± 0.13  -5.93 ± 0.37  57.7 ± 2.6% site 1 | 9 |  | 2.4 | 98 | 234 |
| carazolol | Sigma | 53787 | -6.57 ± 0.03 | 5 | -4.68 ± 0.05^apparent^ | 6 | -5.04 ± 0.08^apparent^ | 5 |  | 78 | 34 | 2.3 |
| carvedilol | Tocris | 2685 | -8.35 ± 0.06 | 12 | -7.84 ± 0.06 | 6 | -7.87 ± 0.12 | 7 |  | 3.2 | 3.0 | 1.1 |
| CGP 12177 | Sigma | C125 | -5.14 ± 0.05 | 6 | Log IC_50_>-4 | 5 | -4.20 ± 0.11 | 5 |  | >14 | 8.7 | >1.6 |
| CGP 20712A | Tocris | 1024 | -4.93 ± 0.10 | 5 | Log IC_50_>-4 | 5 | -4.96 ± 0.07^apparent^ | 5 |  | >8.5 | 1.1 | >9.1 |
| chlorpromazine | Sigma | C8138 | -8.94 ± 0.06 | 5 | -7.84 ± 0.05 | 5 | -8.00 ± 0.08  -5.91 ± 0.20  56.0 ± 5.0% site 1 | 6 |  | 13 | 8.7 | 1.4 |
| citalopram | Tocris | 1427 | -5.95 ± 0.06 | 4 | IC50 ~ -4 | 4 | -4.91 ± 0.11 | 5 |  | >89 | 11 | >8.1 |
| clomipramine | Sigma | C7291 | -8.12 ± 0.10 | 9 | -6.34 ± 0.07 | 9 | -6.15 ± 0.09 | 5 |  | 60 | 93 | 1.5 |
| clozapine | Sigma | 16305 | -8.27 ± 0.04 | 5 | -7.39 ± 0.07 | 5 | -6.41 ± 0.05 | 5 |  | 7.6 | 72 | 9.5 |
| cyanopindolol | Tocris | 0993 | -5.59 ± 0.05 | 8 | -4.91 ± 0.09 | 7 | -5.40 ± 0.07 | 5 |  | 4.8 | 1.5 | 3.1 |
| cyclazosin | Sigma | C247 | -8.89 ± 0.06 | 7 | -8.68 ± 0.08 | 5 | -9.87 ± 0.06  -7.44 ± 0.10  56.8 ± 3.4% site 1 | 7 |  | 1.6 | 9.5 | 15 |
| desipramine | Sigma | D3900 | -7.07 ± 0.05 | 6 | -5.57 ± 0.05 | 5 | -5.46 ± 0.07 | 5 |  | 32 | 41 | 1.3 |
| dibenamine | Sigma | 291366 | -7.91 ± 0.06  -5.32 ± 0.08  83.0 ± 1.8% site 1 | 15 | -6.57 ± 0.07  -4.66 ± 0.06  67.6 ± 2.6% site 1 | 14 | -7.37 ± 0.15  -5.00 ± 0.14  47.8 ± 3.2% site 1 | 9 |  | 22 | 3.5 | 6.3 |
| domperidone | Sigma | D122 | -6.85 ± 0.12 | 6 | -5.50 ± 0.05 | 5 | -5.98 ± 0.05 | 5 |  | 22 | 7.4 | 3.0 |
| dosulepin | EPRS | D2962000 | -7.11 ± 0.04 | 5 | -5.28 ± 0.11 | 7 | -5.58 ± 0.05 | 5 |  | 68 | 34 | 2.0 |
| doxepin | Tocris | 0508 | -7.74 ± 0.04 | 5 | -6.18 ± 0.03 | 5 | -6.27 ± 0.11 | 6 |  | 36 | 30 | 1.2 |
| doxazosin | Sigma | D985 | -8.58 ± 0.09 | 6 | -8.46 ± 0.05 | 8 | -8.33 ± 0.13 | 11 |  | 1.3 | 1.8 | 1.3 |
| duloxetine | sigma | PHR1865 | -5.65 ± 0.05 | 5 | -4.71 ± 0.03^apparent^ | 5 | -5.58 ± 0.12 | 7 |  | 8.7 | 1.2 | 7.4 |
| efaroxan | Tocris | 0792 | -5.47 ± 0.03 | 5 | -4.27 ± 0.07 | 5 | -4.97 ± 0.06 | 5 |  | 16 | 3.2 | 5.0 |
| fluoxetine | Sellakchem | S1333 | -5.45 ± 0.04 | 5 | -4.41 ± 0.06 | 5 | -4.90 ± 0.13 | 5 |  | 11 | 3.5 | 3.1 |
| flupenthixol | Tocris | 4057 | -8.35 ± 0.05 | 5 | -7.47 ± 0.07 | 5 | -6.96 ± 0.12 | 7 |  | 7.6 | 25 | 3.2 |
| fluvoxamine | Sellakchem | S1336 | -6.10 ± 0.03 | 5 | Log IC_50_>-4 | 5 | -4.97 ± 0.03 | 5 |  | >126 | 14 | >9.3 |
| haloperidol | Sigma | H1512 | -7.70 ± 0.03 | 5 | -7.21 ± 0.07 | 6 | -6.42 ± 0.06 | 5 |  | 3.1 | 19 | 6.2 |
| HEAT | Tocris | 0535 | -8.57 ± 0.06 | 5 | -8.04 ± 0.04 | 5 | -8.11 ± 0.18  -5.15 ± 0.26  64.1 ± 4.1% site 1 | 8 |  | 3.4 | 2.9 | 1.2 |
| ICI118551 | Sigma | I127 | -5.23 ± 0.03 | 5 | -4.20 ± 0.06^apparent^ | 5 | -4.96 ± 0.03 | 5 |  | 11 | 1.9 | 5.8 |
| idazoxan | Sigma | I6138 | -5.67 ± 0.07 | 5 | -4.88 ± 0.03 | 5 | -5.23 ± 0.11 | 5 |  | 6.2 | 2.8 | 2.2 |
| ifenprodil | Sellakchem | S4091 | -7.66 ± 0.11 | 9 | -6.49 ± 0.07 | 6 | -8.12 ± 0.18  -6.05 ± 0.13  48.8 ± 4.5% site 1 | 8 |  | 15 | 2.9 | 43 |
| imipramine | Sigma | I0899 | -7.47 ± 0.04 | 6 | -5.76 ± 0.05 | 6 | -5.89 ± 0.05 | 5 |  | 51 | 38 | 1.3 |
| imiloxan | Sigma | I9531 | -4.60 ± 0.05 | 5 | Log IC_50_>-4 | 5 | -5.02 ± 0.07 | 5 |  | >4.0 | 2.6 | >10 |
| indoramin | Sigma | I2909 | -8.43 ± 0.07 | 5 | -6.82 ± 0.04 | 5 | -6.29 ± 0.07 | 5 |  | 41 | 138 | 3.4 |
| JP1302 | Tocris | 2666 | -6.21 ± 0.04 | 5 | -5.46 ± 0.02 | 5 | -5.58 ± 0.09 | 5 |  | 5.6 | 4.3 | 1.3 |
| labetolol | Sigma | L1011 | -7.33 ± 0.04 | 7 | -5.91 ± 0.03 | 7 | -6.12 ± 0.07 | 6 |  | 26 | 16 | 1.6 |
| lisuride | Tocris | 4052 | -7.94 ± 0.06 | 5 | -6.07 ± 0.04 | 5 | -6.93 ± 0.11 | 7 |  | 74 | 10 | 7.2 |
| lurasidone | Sellakchem | S3044 | -7.80 ± 0.11 | 5 | -7.17 ± 0.09 | 5 | -8.19 ± 0.10  -5.92 ± 0.06  24.8 ± 3.1% site 1 | 7 |  | 4.3 | 2.5 | 10 |
| lofepramine | Tocris | 2545 | -6.94 ± 0.06 | 6 | -5.44 ± 0.07 | 6 | -5.37 ± 0.04 | 5 |  | 32 | 37 | 1.2 |
| 5-methyl-urapidil | Sigma | H101 | -8.23 ± 0.05 | 5 | -6.06 ± 0.04 | 5 | -5.61 ± 0.07 | 5 |  | 148 | 417 | 2.8 |
| MK-912 | Sigma | M7065 | -6.76 ± 0.03 | 5 | -5.46 ± 0.05 | 5 | -7.30 ± 0.16  -5.50 ± 0.25  61.2 ± 5.5% site 1 | 7 |  | 20 | 3.5 | 69 |
| mirtazepine | Tocris | 2018 | -6.36 ± 0.02 | 5 | -5.36 ± 0.03 | 5 | -5.94 ± 0.05 | 5 |  | 10 | 2.6 | 3.8 |
| 2-MPMDQ | Tocris | 0661 | -9.06 ± 0.07 | 6 | -7.37 ± 0.04 | 6 | -9.01 ± 0.16  -5.66 ± 0.29  64.0 ± 2.1% site 1 | 8 |  | 49 | 1.1 | 44 |
| 3-MPPI | Tocris | 0581 | -9.57 ± 0.06 | 6 | -8.59 ± 0.03 | 6 | -9.76 ± 0.15  -6.93 ± 0.17  66.7 ± 3.4% site 1 | 7 |  | 9.5 | 1.5 | 15 |
| naftopidil | Tocris | 0597 | -7.97 ± 0.03 | 6 | -6.82 ± 0.06 | 6 | -7.06 ± 0.11 | 7 |  | 14 | 8.1 | 1.7 |
| niguldipine | Tocris | 1123 | -9.24 ± 0.11 | 9 | -6.33 ± 0.08 | 5 | -5.92 ± 0.06 | 6 |  | 813 | 2089 | 2.6 |
| norclomipramine | sigma | N1280 | -7.52 ± 0.08 | 11 | -5.84 ± 0.04 | 12 | -5.84 ± 0.06 | 5 |  | 48 | 48 | 1.0 |
| nortriptyline | Sigma | N7261 | -7.74 ± 0.03 | 6 | -6.07 ± 0.07 | 5 | -5.81 ± 0.05 | 5 |  | 47 | 85 | 1.8 |
| olanzapine | Sigma | O1141 | -6.61 ± 0.11 | 7 | -6.00 ± 0.10 | 10 | -5.86 ± 0.06 | 5 |  | 4.1 | 5.6 | 1.4 |
| 2-PMDQ | Tocris | 0627 | -8.19 ± 0.09 | 5 | -6.95 ± 0.05 | 6 | -8.42 ± 0.12  -5.61 ± 0.12  57.6 ± 2.8% site 1 | 9 |  | 17 | 1.7 | 30 |
| paliperidone | Sellakchem | S1724 | -8.36 ± 0.09 | 5 | -7.36 ± 0.08 | 5 | -7.47 ± 0.10  -5.57 ± 0.21  57.6 ± 4.9% site 1 | 6 |  | 10 | 7.8 | 1.3 |
| paroxetine | Sellakcham | S3005 | -5.59 ± 0.09 | 5 | Log IC_50_>-5 | 5 | -5.63 ± 0.13 | 5 |  | >3.9 | 1.1 | >4.3 |
| perphenazine | Sellakchem | S4731 | -8.15 ± 0.09 | 5 | -7.43 ± 0.08 | 5 | -7.86 ± 0.10  -6.03 ± 0.19  53.3 ± 4.1% site 1 | 5 |  | 5.2 | 1.9 | 2.7 |
| phenoxybenzamine | Sigma | B019 | -8.45 ± 0.12  -6.02 ± 0.08  77.7 ± 5.2% site 1 | 12 | -7.69 ± 0.06  -5.57 ± 0.06  67.5 ± 2.5% site 1 | 13 | -8.43 ± 0.19  -5.42 ± 0.08  39.1 ± 2.0% site 1 | 10 |  | 5.8 | 1.0 | 5.5 |
| phentolamine | Sigma | P7547 | -8.15 ± 0.08 | 8 | -6.55 ± 0.05 | 5 | -6.84 ± 0.11  -4.64 ± 0.14  60.5 ± 3.8% site 1 | 6 |  | 40 | 20 | 1.9 |
| pimozide | Tocris | 0937 | -7.44 ± 0.16 | 5 | -6.79 ± 0.05 | 5 | -5.95 ± 0.08 | 6 |  | 4.5 | 31 | 6.9 |
| prazosin | Tocris | 0623 | -9.07 ± 0.04 | 8 | -8.74 ± 0.06 | 8 | -9.07 ± 0.23 | 10 |  | 2.1 | 1.0 | 1.3 |
| prochlorperazine | Sigma | P9178 | -7.61 ± 0.12 | 5 | -6.88 ± 0.06 | 5 | -6.53 ± 0.13 | 8 |  | 5.4 | 12 | 2.2 |
| promethazine | Sigma | P4651 | -7.00 ± 0.10 | 11 | -6.06 ± 0.05 | 10 | -5.75 ± 0.07 | 5 |  | 8.7 | 18 | 2.0 |
| propranolol | Sigma | P0884 | -4.91 ± 0.02 | 6 | -3.98 ± 0.04^apparent^ | 6 | -4.89 ± 0.09 | 5 |  | 8.5 | 1.0 | 8.1 |
| protriptyline | Sigma | P8813 | -6.67 ± 0.03 | 5 | -5.57 ± 0.02 | 5 | -5.46 ± 0.12 | 5 |  | 13 | 16 | 1.3 |
| quetiapine | Sellakchem | S1763 | -7.89 ± 0.10 | 5 | -7.21 ± 0.04 | 5 | -6.48 ± 0.10 | 5 |  | 4.8 | 26 | 5.4 |
| Rec15-2615 | Tocris | 3284 | -8.26 ± 0.10 | 6 | -7.79 ± 0.09 | 6 | -7.89 ± 0.07 | 7 |  | 3.0 | 2.3 | 1.3 |
| reboxetine | Sellakchem | S3199 | -4.91 ± 0.08 | 5 | Log IC_50_>-4 | 5 | -4.67 ± 0.12 | 5 |  | >8.1 | 1.7 | >4.7 |
| risperidone | Sigma | R3030 | -8.74 ± 0.06 | 7 | -7.77 ± 0.05 | 7 | -7.14 ± 0.07 | 6 |  | 9.3 | 40 | 4.3 |
| RS100329 | Tocris | 1352 | -9.60 ± 0.05 | 10 | -6.67 ± 0.07 | 7 | -7.63 ± 0.17  -5.13 ± 0.15  70.5 ± 3.0% site 1 | 6 |  | 851 | 93 | 9.1 |
| RS17053 | Tocris | 0985 | -8.33 ± 0.09 | 11 | -6.61 ± 0.09 | 11 | -6.84 ± 0.13 | 6 |  | 52 | 31 | 1.7 |
| RS79948 | Tocris | 0987 | -5.75 ± 0.05 | 5 | -4.99 ± 0.05 | 5 | -6.07 ± 0.11 | 6 |  | 5.8 | 2.1 | 12 |
| RX 821002 | Sigma | R9525 | -6.51 ± 0.09 | 6 | -5.46 ± 0.06 | 6 | -5.31 ± 0.13 | 7 |  | 11 | 16 | 1.4 |
| S32212 | Tocris | 4508 | -5.90 ± 0.06 | 5 | -4.92 ± 0.02^apparent^ | 5 | -5.69 ± 0.13 | 5 |  | 9.5 | 1.6 | 5.9 |
| SDZ 21009 | Tocris | 1516 | -5.24 ± 0.07 | 6 | Log IC_50_>-4 | 6 | -5.09 ± 0.18 | 6 |  | >17 | 1.4 | >12 |
| sertindole | Sigma | S8072 | -9.27 ± 0.09 | 9 | -8.28 ± 0.11 | 8 | -6.93 ± 0.12 | 8 |  | 9.8 | 219 | 22 |
| sertraline | Sellakchem | S4053 | -5.72 ± 0.04 | 5 | -5.45 ± 0.05 | 5 | -5.61 ± 0.04 | 5 |  | 1.9 | 1.3 | 1.4 |
| silodosin | Sellakchem | S1613 | -9.61 ± 0.08 | 9 | -6.50 ± 0.09 | 9 | -6.94 ± 0.16 | 9 |  | 1288 | 468 | 2.8 |
| SKF86466 | Tocris | 3866 | -6.06 ± 0.05 | 5 | -4.93 ± 0.05 | 5 | -5.16 ± 0.09 | 5 |  | 13.5 | 7.9 | 1.7 |
| SNAP5089 | Tocris | 2398 | -8.89 ± 0.03 | 6 | -5.64 ± 0.04^apparent^ | 6 | -5.65 ± 0.09^apparent^ | 5 |  | 1778 | 1738 | 1.0 |
| spiroxatrine | Tocris | 0631 | -6.86 ± 0.06 | 5 | -6.41 ± 0.06 | 5 | -7.86 ± 0.13  -5.84 ± 0.31  61.9 ± 4.0% site 1 | 6 |  | 2.8 | 10 | 28 |
| sulpiride | Sigma | S8010 | -4.50 ± 0.07 | 5 | Log IC_50_>-3 | 5 | -3.66 ± 0.09^apparent^ | 5 |  | >31 | 6.9 | >4.6 |
| sunepitron | Sigma | PZ0279 | -5.78 ± 0.06 | 5 | -4.65 ± 0.06 | 5 | -5.33 ± 0.23 | 6 |  | 13 | 2.8 | 4.8 |
| tamsulosin | Sigma | T1330 | -9.67 ± 0.06 | 17 | -8.12 ± 0.04 | 15 | -9.18 ± 0.08  -5.67 ± 0.15  54.6 ± 3.7% site 1 | 13 |  | 35 | 3.1 | 11 |
| terazosin | Tocris | 1506 | -7.93 ± 0.05 | 6 | -7.95 ± 0.05 | 6 | -7.71 ± 0.13 | 7 |  | 1.0 | 1.7 | 1.7 |
| trazodone | Sigma | T6154 | -7.33 ± 0.04 | 6 | -6.56 ± 0.07 | 6 | -6.38 ± 0.15 | 7 |  | 5.9 | 8.9 | 1.5 |
| trifluoperazine | Sigma | T8516 | -7.75 ± 0.03 | 5 | -6.88 ± 0.07 | 5 | -6.36 ± 0.08 | 5 |  | 7.4 | 25 | 3.3 |
| trimipramine | Sigma | T3146 | -7.37 ± 0.08 | 5 | -6.10 ± 0.06 | 5 | -5.99 ± 0.05 | 5 |  | 19 | 24 | 1.3 |
| urapidil | Tocris | 1772 | -7.21 ± 0.02 | 5 | -5.50 ± 0.07 | 7 | -6.37 ± 0.10 | 6 |  | 51 | 6.9 | 7.4 |
| venlafaxime | Sellakchem | S1441 | -3.69 ± 0.02^apparent^ | 5 | No binding to 1mM | 5 | -4.38 ± 0.16 | 7 |  | >4.9 | 4.9 | >24 |
| vortioxetine | Sellakchem | S8021 | -6.32 ± 0.05 | 5 | -5.42 ± 0.02 | 5 | -5.43 ± 0.08 | 5 |  | 7.9 | 7.8 | 1.0 |
| WB4104 | Tocris | 0946 | -9.03 ± 0.04 | 10 | -7.39 ± 0.05 | 7 | -8.63 ± 0.11  -5.96 ± 0.09  59.6 ± 3.2% site 1 | 9 |  | 44 | 2.5 | 17 |
| yohimbine | Sigma | Y3125 | -6.23 ± 0.03 | 5 | -5.44 ± 0.05 | 5 | -6.20 ± 0.08 | 8 |  | 6.2 | 1.1 | 5.8 |
| ziprasidone | Sellakchem | S1444 | -8.73 ± 0.05 | 7 | -7.70 ± 0.07 | 8 | -7.20 ± 0.09 | 5 |  | 11 | 34 | 3.2 |

^apparent^ the maximum concentration of competing ligand inhibited most but not all of specific binding (as in Figure 1e). An IC_50_ was determined by extrapolating the curve assuming that all specific binding would be inhibited if a higher concentration of competing ligand were possible. Thus an apparent K_D_ was calculated.

^early plateau^ bromocriptine did not fully inhibit specific binding at the α1D-adrenoceptor. The inhibition curve reached a plateau of maximal inhibition of binding at 71.0 ± 3.4% inhibition of specific binding (n=9)

**Supplementary Data Table 2**

Affinity (log K_D_ values) of α-antagonists obtained from ^3^H-prazosin whole cell binding to the human α1A, α1B and α1D-adrenoceptors stably expressed in CHO cells. Ligands are arranged in order of α1A-adrenoceptor affinity. Values represent mean ± s.e.mean of n separate experiments. Selectivity ratios are also given where a ratio of 1 demonstrates no selectivity for a given receptor subtype over another.

|  | Log K_D_ values from ^3^H-prazosin whole cell binding | | | | | | | |  | selectivities | | |
| --- | --- | --- | --- | --- | --- | --- | --- | --- | --- | --- | --- | --- |
| ligand | Log K_D_ α1A | n |  | Log K_D_ α1B | n |  | Log K_D_ α1D | n |  | α1A vs α1B | α1A vs α1D | α1B vs α1D |
| tamsulosin | -9.67 ± 0.06 | 17 |  | -8.12 ± 0.04 | 15 | . | -9.18 ± 0.08  -5.67 ± 0.15  54.6 ± 3.7% site 1 | 13 |  | 35 | 3.1 | 11 |
| silodosin | -9.61 ± 0.08 | 9 |  | -6.50 ± 0.09 | 9 |  | -6.94 ± 0.16 | 9 |  | 1288 | 468 | 2.8 |
| RS100329 | -9.60 ± 0.05 | 10 |  | -6.67 ± 0.07 | 7 |  | -7.63 ± 0.17  -5.13 ± 0.15  70.5 ± 3.0% site 1 | 6 |  | 851 | 93 | 9.1 |
| 3-MPPI | -9.57 ± 0.06 | 6 |  | -8.59 ± 0.03 | 6 |  | -9.76 ± 0.15  -6.93 ± 0.17  66.7 ± 3.4% site 1 | 7 |  | 9.5 | 1.5 | 15 |
| ARC239 | -9.35 ± 0.08 | 8 |  | -8.15 ± 0.07 | 9 |  | -8.74 ± 0.12  -5.42 ± 0.21  60.5 ± 1.4% site 1 | 7 |  | 16 | 4.1 | 3.9 |
| sertindole | -9.27 ± 0.09 | 9 |  | -8.28 ± 0.11 | 8 |  | -6.93 ± 0.12 | 8 |  | 9.8 | 219 | 22 |
| niguldipine | -9.24 ± 0.11 | 9 |  | -6.33 ± 0.08 | 5 |  | -5.92 ± 0.06 | 6 |  | 813 | 2089 | 2.6 |
| benoxathian | -9.08 ± 0.05 | 6 |  | -7.32 ± 0.03 | 6 |  | -7.91 ± 0.10  -5.62 ± 0.19  62.5 ± 3.4% site 1 | 7 |  | 58 | 15 | 3.9 |
| prazosin | -9.07 ± 0.04 | 8 |  | -8.74 ± 0.06 | 8 |  | -9.07 ± 0.23 | 10 |  | 2.1 | 1.0 | 2.1 |
| 2-MPMDQ | -9.06 ± 0.07 | 6 |  | -7.37 ± 0.04 | 6 |  | -9.01 ± 0.16  -5.66 ± 0.29  64.0 ± 2.1% site1 | 8 |  | 49 | 1.1 | 44 |
| WB4104 | -9.03 ± 0.04 | 10 |  | -7.39 ± 0.05 | 7 |  | -8.63 ± 0.11  -5.96 ± 0.09  59.6 ± 3.2% site 1 | 9 |  | 44 | 2.5 | 17 |
| chlorpromazine | -8.94 ± 0.06 | 5 |  | -7.84 ± 0.05 | 5 |  | -8.00 ± 0.08  -5.91 ± 0.20  56.0 ± 5.0% site 1 | 6 |  | 13 | 8.7 | 1.4 |
| cyclazosin | -8.89 ± 0.06 | 7 |  | -8.68 ± 0.08 | 5 |  | -9.87 ± 0.06  -7.44 ± 0.10  56.8 ± 3.4% site 1 | 7 |  | 1.6 | 9.5 | 15 |
| SNAP5089 | -8.89 ± 0.03 | 6 |  | -5.64 ± 0.04^apparent^ | 6 |  | -5.65 ± 0.09^apparent^ | 5 |  | 1778 | 1738 | 1.0 |
| risperidone | -8.74 ± 0.06 | 7 |  | -7.77 ± 0.05 | 7 |  | -7.14 ± 0.07 | 6 |  | 9.3 | 40 | 4.3 |
| bromocriptine | -8.73 ± 0.06 | 5 |  | -7.96 ± 0.07 | 5 |  | -7.31 ± 0.15^early plateau^ | 9 |  | 5.9 | 26 | 4.5 |
| ziprasidone | -8.73 ± 0.05 | 7 |  | -7.70 ± 0.07 | 8 |  | -7.20 ± 0.09 | 5 |  | 11 | 34 | 3.2 |
| doxazosin | -8.58 ± 0.09 | 6 |  | -8.46 ± 0.05 | 8 |  | -8.33 ± 0.13 | 11 |  | 1.3 | 1.8 | 1.3 |
| HEAT | -8.57 ± 0.06 | 5 |  | -8.04 ± 0.04 | 5 |  | -8.11 ± 0.18  -5.15 ± 0.26  64.1 ± 4.1% site 1 | 8 |  | 3.4 | 2.9 | 1.2 |
| phenoxybenzamine | -8.45 ± 0.12  -6.02 ± 0.08  77.7 ± 5.2% site 1 | 12 |  | -7.69 ± 0.06  -5.57 ± 0.06  67.5 ± 2.5% site 1 | 13 |  | -8.43 ± 0.19  -5.42 ± 0.08  39.1 ± 2.0% site 1 | 10 |  | 5.8 | 1.0 | 5.5 |
| indoramin | -8.43 ± 0.07 | 5 |  | -6.82 ± 0.04 | 5 |  | -6.29 ± 0.07 | 5 |  | 41 | 138 | 3.4 |
| paliperidone | -8.36 ± 0.09 | 5 |  | -7.36 ± 0.08 | 5 |  | -7.47 ± 0.10  -5.57 ± 0.21  57.6 ± 4.9% site 1 | 6 |  | 10 | 7.8 | 1.3 |
| flupenthixol | -8.35 ± 0.05 | 5 |  | -7.47 ± 0.07 | 5 |  | -6.96 ± 0.12 | 7 |  | 7.6 | 25 | 3.2 |
| carvedilol | -8.35 ± 0.06 | 12 |  | -7.84 ± 0.06 | 6 |  | -7.87 ± 0.12 | 7 |  | 3.2 | 3.0 | 1.1 |
| RS17053 | -8.33 ± 0.09 | 11 |  | -6.61 ± 0.09 | 11 |  | -6.84 ± 0.13 | 6 |  | 52 | 31 | 1.7 |
| clozapine | -8.27 ± 0.04 | 5 |  | -7.39 ± 0.07 | 5 |  | -6.41 ± 0.05 | 5 |  | 7.6 | 72 | 9.5 |
| Rec15-2615 | -8.26 ± 0.10 | 6 |  | -7.79 ± 0.09 | 6 |  | -7.89 ± 0.07 | 7 |  | 3.0 | 2.3 | 1.3 |
| 5-methyl-urapidil | -8.23 ± 0.05 | 5 |  | -6.06 ± 0.04 | 5 |  | -5.61 ± 0.07 | 5 |  | 148 | 417 | 2.8 |
| 2-PMDQ | -8.19 ± 0.09 | 5 |  | -6.95 ± 0.05 | 6 |  | -8.42 ± 0.12  -5.61 ± 0.12  57.6 ± 2.8% site 1 | 9 |  | 17 | 1.7 | 30 |
| amitriptyline | -8.19 ± 0.02 | 9 |  | -6.22 ± 0.05 | 9 |  | -6.25 ± 0.05 | 5 |  | 93 | 87 | 1.1 |
| perphenazine | -8.15 ± 0.09 | 5 |  | -7.43 ± 0.08 | 5 |  | -7.86 ± 0.10  -6.03 ± 0.19  53.3 ± 4.1% site 1 | 5 |  | 5.2 | 1.9 | 2.7 |
| phentolamine | -8.15 ± 0.08 | 8 |  | -6.55 ± 0.05 | 5 |  | -6.84 ± 0.11  -4.64 ± 0.14  60.5 ± 3.8% site 1 | 6 |  | 40 | 20 | 1.9 |
| clomipramine | -8.12 ± 0.10 | 9 |  | -6.34 ± 0.07 | 9 |  | -6.15 ± 0.09 | 5 |  | 60 | 93 | 1.5 |
| naftopidil | -7.97 ± 0.03 | 6 |  | -6.82 ± 0.06 | 6 |  | -7.06 ± 0.11 | 7 |  | 14 | 8.1 | 1.7 |
| lisuride | -7.94 ± 0.06 | 5 |  | -6.07 ± 0.04 | 5 |  | -6.93 ± 0.11 | 7 |  | 74 | 10 | 7.2 |
| terazosin | -7.93 ± 0.05 | 6 |  | -7.95 ± 0.05 | 6 |  | -7.71 ± 0.13 | 7 |  | 1.0 | 1.7 | 1.7 |
| dibenamine | -7.91 ± 0.06  -5.32 ± 0.08  83.0 ± 1.8% site 1 | 15 |  | -6.57 ± 0.07  -4.66 ± 0.06  67.6 ± 2.6% site 1 | 14 |  | -7.37 ± 0.15  -5.00 ± 0.14  47.8 ± 3.2% site 1 | 9 |  | 22 | 3.5 | 6.3 |
| quetiapine | -7.89 ± 0.10 | 5 |  | -7.21 ± 0.04 | 5 |  | -6.48 ± 0.10 | 5 |  | 4.8 | 26 | 5.4 |
| alfuzosin | -7.82 ± 0.11 | 8 |  | -7.56 ± 0.08 | 6 |  | -7.66 ± 0.11 | 6 |  | 1.8 | 1.4 | 1.3 |
| lurasidone | -7.80 ± 0.11 | 5 |  | -7.17 ± 0.09 | 5 |  | -8.19 ± 0.10  -5.92 ± 0.06  24.8 ± 3.1% site 1 | 7 |  | 4.3 | 2.5 | 10 |
| trifluoperazine | -7.75 ± 0.03 | 5 |  | -6.88 ± 0.07 | 5 |  | -6.36 ± 0.08 | 5 |  | 7.4 | 25 | 3.3 |
| doxepin | -7.74 ± 0.04 | 5 |  | -6.18 ± 0.03 | 5 |  | -6.27 ± 0.11 | 6 |  | 36 | 30 | 1.2 |
| nortriptyline | -7.74 ± 0.03 | 6 |  | -6.07 ± 0.07 | 5 |  | -5.81 ± 0.05 | 5 |  | 47 | 85 | 1.8 |
| haloperidol | -7.70 ± 0.03 | 5 |  | -7.21 ± 0.07 | 6 |  | -6.42 ± 0.06 | 5 |  | 3.1 | 19 | 6.2 |
| ifenprodil | -7.66 ± 0.11 | 9 |  | -6.49 ± 0.07 | 6 |  | -8.12 ± 0.18  -6.05 ± 0.13  48.8 ± 4.5% site 1 | 8 |  | 15 | 2.9 | 43 |
| prochlorperazine | -7.61 ± 0.12 | 5 |  | -6.88 ± 0.06 | 5 |  | -6.53 ± 0.13 | 8 |  | 5.4 | 12 | 2.2 |
| bucindolol | -7.57 ± 0.07 | 5 |  | -6.46 ± 0.04 | 5 |  | -6.45 ± 0.09 | 5 |  | 13 | 13 | 1.0 |
| norclomipramine | -7.52 ± 0.08 | 11 |  | -5.84 ± 0.04 | 12 |  | -5.84 ± 0.06 | 5 |  | 48 | 48 | 1.0 |
| imipramine | -7.47 ± 0.04 | 6 |  | -5.76 ± 0.05 | 6 |  | -5.89 ± 0.05 | 5 |  | 51 | 38 | 1.3 |
| pimozide | -7.44 ± 0.16 | 5 |  | -6.79 ± 0.05 | 5 |  | -5.95 ± 0.08 | 6 |  | 4.5 | 31 | 6.9 |
| trimipramine | -7.37 ± 0.08 | 5 |  | -6.10 ± 0.06 | 5 |  | -5.99 ± 0.05 | 5 |  | 19 | 24 | 1.3 |
| trazodone | -7.33 ± 0.04 | 6 |  | -6.56 ± 0.07 | 6 |  | -6.38 ± 0.15 | 7 |  | 5.9 | 8.9 | 1.5 |
| labetolol | -7.33 ± 0.04 | 7 |  | -5.91 ± 0.03 | 7 |  | -6.12 ± 0.07 | 6 |  | 26 | 16 | 1.6 |
| aripiprazole | -7.32 ± 0.07 | 6 |  | -6.69 ± 0.03 | 6 |  | -6.15 ± 0.11 | 5 |  | 4.3 | 15 | 3.5 |
| urapidil | -7.21 ± 0.02 | 5 |  | -5.50 ± 0.07 | 7 |  | -6.37 ± 0.10 | 6 |  | 51 | 6.9 | 7.4 |
| dosulepin | -7.11 ± 0.04 | 5 |  | -5.28 ± 0.11 | 7 |  | -5.58 ± 0.05 | 5 |  | 68 | 34 | 2.0 |
| desipramine | -7.07 ± 0.05 | 6 |  | -5.57 ± 0.05 | 5 |  | -5.46 ± 0.07 | 5 |  | 32 | 41 | 1.3 |
| promethazine | -7.00 ± 0.10 | 11 |  | -6.06 ± 0.05 | 10 |  | -5.75 ± 0.07 | 5 |  | 8.7 | 18 | 2.0 |
| lofepramine | -6.94 ± 0.06 | 6 |  | -5.44 ± 0.07 | 6 |  | -5.37 ± 0.04 | 5 |  | 32 | 37 | 1.2 |
| spiroxatrine | -6.86 ± 0.06 | 5 |  | -6.41 ± 0.06 | 5 |  | -7.86 ± 0.13  -5.84 ± 0.31  61.9 ± 4.0%site 1 | 6 |  | 2.8 | 10 | 28 |
| domperidone | -6.85 ± 0.12 | 6 |  | -5.50 ± 0.05 | 5 |  | -5.98 ± 0.05 | 5 |  | 22 | 7.4 | 3.0 |
| MK-912 | -6.76 ± 0.03 | 5 |  | -5.46 ± 0.05 | 5 |  | -7.30 ± 0.16  -5.50 ± 0.25  61.2 ± 5.5% site 1 | 7 |  | 20 | 3.5 | 69 |
| protriptyline | -6.67 ± 0.03 | 5 |  | -5.57 ± 0.02 | 5 |  | -5.46 ± 0.12 | 5 |  | 13 | 16 | 1.3 |
| BMY7378 | -6.61 ± 0.05 | 5 |  | -6.23 ± 0.05 | 6 |  | -8.60 ± 0.13  -5.93 ± 0.37  57.7 ± 2.6% site 1 | 9 |  | 2.4 | 98 | 234 |
| olanzapine | -6.61 ± 0.11 | 7 |  | -6.00 ± 0.10 | 10 |  | -5.86 ± 0.06 | 5 |  | 4.1 | 5.6 | 1.4 |
| A80426 | -6.57 ± 0.05 | 5 |  | -6.08 ± 0.02 | 5 |  | -6.07 ± 0.07 | 5 |  | 3.1 | 3.1 | 1.0 |
| carazolol | -6.57 ± 0.03 | 5 |  | -4.68 ± 0.05^apparent^ | 6 |  | -5.04 ± 0.08^apparent^ | 5 |  | 78 | 34 | 2.3 |
| RX821002 | -6.51 ± 0.09 | 6 |  | -5.46 ± 0.06 | 6 |  | -5.31 ± 0.13 | 7 |  | 11 | 16 | 1.4 |
| AH11110A | -6.48 ± 0.03 | 5 |  | -5.65 ± 0.09 | 5 |  | -4.98 ± 0.06 | 5 |  | 6.8 | 32 | 4.7 |
| mirtazepine | -6.36 ± 0.02 | 5 |  | -5.36 ± 0.03 | 5 |  | -5.94 ± 0.05 | 5 |  | 10 | 2.6 | 3.8 |
| vortioxetine | -6.32 ± 0.05 | 5 |  | -5.42 ± 0.02 | 5 |  | -5.43 ± 0.08 | 5 |  | 7.9 | 7.8 | 1.0 |
| yohimbine | -6.23 ± 0.03 | 5 |  | -5.44 ± 0.05 | 5 |  | -6.20 ± 0.08 | 8 |  | 6.2 | 1.1 | 5.8 |
| JP1302 | -6.21 ± 0.04 | 5 |  | -5.46 ± 0.02 | 5 |  | -5.58 ± 0.09 | 5 |  | 5.6 | 4.3 | 1.3 |
| fluvoxamine | -6.10 ± 0.03 | 5 |  | Log IC_50_>-4 | 5 |  | -4.97 ± 0.03 | 5 |  | >126 | 14 | >9.3 |
| SKF86466 | -6.06 ± 0.05 | 5 |  | -4.93 ± 0.05 | 5 |  | -5.16 ± 0.09 | 5 |  | 13 | 7.9 | 1.7 |
| atipamezole | -5.99 ± 0.03 | 5 |  | -4.68 ± 0.08 | 6 |  | -5.33 ± 0.04 | 5 |  | 20 | 4.6 | 4.5 |
| citalopram | -5.95 ± 0.06 | 4 |  | IC50 ~ -4 | 4 |  | -4.91 ± 0.11 | 5 |  | >89 | 11 | >8.1 |
| BRL44408 | -5.92 ± 0.09 | 9 |  | -4.68 ± 0.07 | 9 |  | -5.06 ± 0.05 | 5 |  | 17 | 7.2 | 2.4 |
| S32212 | -5.90 ± 0.06 | 5 |  | -4.92 ± 0.02^apparent^ | 5 |  | -5.69 ± 0.13 | 5 |  | 9.5 | 1.6 | 5.9 |
| sunepitron | -5.78 ± 0.06 | 5 |  | -4.65 ± 0.06 | 5 |  | -5.33 ± 0.23 | 6 |  | 13 | 2.8 | 4.8 |
| RS79948 | -5.75 ± 0.05 | 5 |  | -4.99 ± 0.05 | 5 |  | -6.07 ± 0.11 | 6 |  | 5.8 | 2.1 | 12 |
| sertraline | -5.72 ± 0.04 | 5 |  | -5.45 ± 0.05 | 5 |  | -5.61 ± 0.04 | 5 |  | 1.9 | 1.3 | 1.4 |
| idazoxan | -5.67 ± 0.07 | 5 |  | -4.88 ± 0.03 | 5 |  | -5.23 ± 0.11 | 5 |  | 6.2 | 2.8 | 2.2 |
| duloxetine | -5.65 ± 0.05 | 5 |  | -4.71 ± 0.03^apparent^ | 5 |  | -5.58 ± 0.12 | 7 |  | 8.7 | 1.2 | 7.4 |
| cyanopindolol | -5.59 ± 0.05 | 8 |  | -4.91 ± 0.09 | 7 |  | -5.40 ± 0.07 | 5 |  | 4.8 | 1.5 | 3.1 |
| paroxetine | -5.59 ± 0.09 | 5 |  | Log IC_50_>-5 | 5 |  | -5.63 ± 0.13 | 5 |  | >3.9 | 1.1 | >4.3 |
| amitraz | -5.52 ± 0.05 | 5 |  | Log IC_50_>-4 | 6 |  | -5.08 ± 0.05 | 5 |  | >33 | 2.8 | >12 |
| efaroxan | -5.47 ± 0.03 | 5 |  | -4.27 ± 0.07 | 5 |  | -4.97 ± 0.06 | 5 |  | 16 | 3.2 | 5.0 |
| fluoxetine | -5.45 ± 0.04 | 5 |  | -4.41 ± 0.06 | 5 |  | -4.90 ± 0.13 | 5 |  | 11 | 3.5 | 3.1 |
| SDZ21009 | -5.24 ± 0.07 | 6 |  | Log IC_50_>-4 | 6 |  | -5.09 ± 0.18 | 6 |  | >17 | 1.4 | >12 |
| ICI118551 | -5.23 ± 0.03 | 5 |  | -4.20 ± 0.06^apparent^ | 5 |  | -4.96 ± 0.03 | 5 |  | 11 | 1.9 | 5.8 |
| anisodamine | -5.21 ± 0.03 | 5 |  | -3.45 ± 0.04^apparent^ | 5 |  | -4.21 ± 0.05 | 5 |  | 58 | 10 | 5.8 |
| CGP 12177 | -5.14 ± 0.05 | 6 |  | Log IC_50_>-4 | 5 |  | -4.20 ± 0.11 | 5 |  | >14 | 8.7 | >1.6 |
| amisulpiride | -5.05 ± 0.04 | 5 |  | No binding to 100µM | 5 |  | -4.55 ± 0.08^apparent^ | 5 |  | 11 | 3.2 | 3.5 |
| CGP 20712A | -4.93 ± 0.10 | 5 |  | Log IC_50_>-4 | 5 |  | -4.96 ± 0.07^apparent^ | 5 |  | >8.5 | 1.1 | >9.1 |
| propranolol | -4.91 ± 0.02 | 6 |  | -3.98 ± 0.04 | 6 |  | -4.89 ± 0.09 | 5 |  | 8.5 | 1.0 | 8.1 |
| reboxetine | -4.91 ± 0.08 | 5 |  | Log IC_50_>-4 | 5 |  | -4.67 ± 0.12 | 5 |  | >8.1 | 1.7 | >4.7 |
| imiloxan | -4.60 ± 0.05 | 5 |  | Log IC_50_>-4 | 5 |  | -5.02 ± 0.07 | 5 |  | >4.0 | 2.6 | >10 |
| agomelatine | -4.57 ± 0.11^apparent^ | 5 |  | No binding to 100µM | 5 |  | Log IC_50_>-4.5 | 6 |  | >3.7 | 1.1 | >3.2 |
| sulpiride | -4.50 ± 0.07 | 5 |  | Log IC_50_>-3 | 5 |  | -3.66 ± 0.09^apparent^ | 5 |  | >32 | 6.9 | >4.6 |
| venlafaxime | -3.69 ± 0.02^apparent^ | 5 |  | No binding to 1mM | 5 |  | -4.38 ± 0.16 | 7 |  | >4.9 | 4.9 | >24 |

^apparent^ the maximum concentration of competing ligand inhibited most but not all of specific binding (as in Figure 1e). An IC_50_ was determined by extrapolating the curve assuming that all specific binding would be inhibited if a higher concentration of competing ligand were possible. Thus an apparent K_D_ was calculated.

^early plateau^ bromocriptine did not fully inhibit specific binding at the α1D-adrenoceptor. The inhibition curve reached a plateau of maximal inhibition of binding at 71.0 ± 3.4% inhibition of specific binding (n=9)
